# Pulmonary Tuberculosis Detection with MiniDock MTB Using Swab Samples

**DOI:** 10.1056/NEJMoa2509761

**Published:** 2026-04-30

**Authors:** Seda Yerlikaya, Masuzyo Chirwa, Bukola Adije, Maria del Mar Castro, Huy Ha, Midori Kato-Maeda, Esther Kisakye, Danaida Marcelo, Tessa Mochizuki, Loren Rockman, Amy Steadman, Balamugesh Thangakunam, John Samson Bimba, Devasahayam J Christopher, Monde Muyoyeta, Ha Phan, Grant Theron, Charles Yu, Kristin Kremer, Patrick PJ Phillips, Payam Nahid, Claudia M. Denkinger, Adithya Cattamanchi, Alfred Andama

**Affiliations:** 1 Department of Infectious Diseases and Tropical Medicine, Heidelberg University Hospital and Faculty of Medicine, Heidelberg University, Heidelberg, Germany; 2 German Center for Infection Research (DZIF), Partner Site Heidelberg University Hospital, Heidelberg, Germany; 3 Centre for Infectious Disease Research in Zambia (CIDRZ), Lusaka, Zambia; 4 Zankli Research Centre, Bingham University Karu, Abuja, Nigeria; 5 Hanoi Lung Hospital, Center for Promotion of Advancement of Society, Hanoi, Vietnam; 6 Division of Pulmonary and Critical Care Medicine, University of California San Francisco, San Francisco, California, USA; 7 UCSF Center for Tuberculosis, Institute for Global Health Sciences, University of California San Francisco, San Francisco, California, USA; 8 World Alliance for Lung and Intensive Care Medicine in Uganda (WALIMU), Kampala, Uganda; 9 De La Salle Medical and Health Sciences Institute, Dasmariñas City, Cavite, Philippines; 10 DSI-NRF Centre of Excellence for Biomedical Tuberculosis Research, South African Medical Research Council Centre for Tuberculosis Research, Division of Molecular Biology and Human Genetics, Faculty of Medicine and Health Sciences, Stellenbosch University, South Africa; 11 Global Health Labs, Inc., Bellevue, WA USA; 12 Department of Pulmonary Medicine, Christian Medical College, Vellore, Tamil Nadu, India; 13 Division of TB Elimination & Health Systems Innovations, KNCV Tuberculosis Foundation, 2516 AB, The Hague, the Netherlands; 14 Division of Pulmonary Diseases and Critical Care Medicine, University of California Irvine, Orange, California, USA; 15 Department of Medicine, Makerere University College of Health Sciences, Kampala, Uganda

## Abstract

**Background:**

Improved tuberculosis (TB) diagnostics suitable for peripheral health centers are essential for reducing the persistent gap between estimated and notified cases. We evaluated the diagnostic accuracy and usability of a nucleic acid amplification-based device, MiniDock MTB, which uses swabs to test for pulmonary TB.

**Methods:**

We conducted a prospective, cross-sectional study at outpatient centers in India, Nigeria, South Africa, the Philippines, Uganda, Vietnam, and Zambia. Participants aged ≥12 years with presumptive pulmonary TB were enrolled between September 12, 2024 and March 31, 2025. MiniDock MTB was performed on sputum and tongue swabs. Diagnostic accuracy was evaluated against a sputum culture-based reference and compared to sputum smear microscopy and Xpert MTB/RIF Ultra (Cepheid, USA). Usability was assessed using the System Usability Scale and direct observation.

**Results:**

Among 1380 participants, 255 (18.6%) were living with HIV and 226 (17.4%) had culture-confirmed TB. MiniDock MTB sensitivity was 85.7% (95% CI, 80.4–90.0) on sputum and 79.6% (95% CI, 73.8–84.7) on tongue swabs; specificity was ≥97.5% for both. Sputum MiniDock MTB matched sputum Xpert Ultra sensitivity (difference: −2.8% [95% CI −6.0–0.5]). MiniDock MTB outperformed smear microscopy (sputum: +24.3% [95% CI 17.9–30.7], tongue swab: +18.3% [95% CI 12.0–24.7]). The median System Usability Scale score was 75 (IQR: 65–80), indicating good usability. No adverse events related to the index test were reported.

**Conclusions:**

MiniDock MTB meets WHO accuracy targets for sputum- and non-sputum-based TB testing.

Tuberculosis (TB) remains a leading cause of infectious disease-related mortality worldwide, making timely diagnosis essential for effective treatment and control.^1^ However, access to rapid, accurate, and user-friendly diagnostics at or near the point-of-care (POC) is still limited in many high-burden settings.

MTB Nucleic Acid Test Card (MiniDock MTB Test; Guangzhou Pluslife Biotech, China) is a qualitative molecular test designed for detecting *Mycobacterium tuberculosis* complex (MTBC) using the Pluslife Integrated Nucleic Acid Testing Device (MiniDock PM001 Ultra), a low cost, portable, battery-operated isothermal platform. The test employs RNase hybridization–assisted amplification (RHAM), combining loop-mediated isothermal amplification (LAMP) with RNase HII–mediated signal detection in a single reaction, producing visual results in 12 to 25 minutes. It targets conserved regions of the IS*6110* and *gyr*B genes and includes an internal control to verify sample adequacy and amplification integrity. Sample preparation is performed using Pluslife Thermolyse, which combines automated bead-beating (3000 rpm) and thermal lysis (75°C for 5 minutes) to process sputum (using swab swirled in sputum) or tongue swab samples.

In a multicenter pilot study, a prototype test demonstrated sensitivity of 89.9% (95% CI, 80.2–95.8) for sputum and 85.7% (95% CI, 75.3–92.9) for tongue swabs, with >98% specificity for both, exceeding World Health Organization (WHO) target product profile minimum accuracy targets for near-POC TB diagnostics. ^2,3^

We conducted an evaluation of the design-locked version of the MiniDock MTB Test intended for clinical use, assessing its diagnostic accuracy and usability across seven high-burden countries. Diagnostic performance was assessed against a microbiological reference standard and compared to sputum smear microscopy and sputum Xpert MTB/RIF Ultra (Ultra; Cepheid, USA). Usability was assessed among healthcare workers (HCWs) involved in routine sample collection and TB testing at participating sites.

## Methods

### Study Design and Setting:

This prospective, cross-sectional, multicenter diagnostic accuracy study was conducted in outpatient clinics in India, Nigeria, the Philippines, South Africa, Uganda, Vietnam, and Zambia as part of the Rapid Research in Diagnostics Development for TB Network (R2D2 TB Network) and the Assessing Diagnostics At Point-of-care for Tuberculosis (ADAPT) studies (Table S1).

### Study Participants:

We screened consecutive individuals presenting to participating health centers between September 12, 2024 and March 31, 2025 (Table S1), and enrolled those aged 12 years or older who met pre-specified eligibility criteria. Eligible participants had either a new or worsening cough lasting at least two weeks, or at least one TB risk factor combined with a positive result on a WHO-recommended TB screening test (see Supplementary Methods and protocol for details, both available at NEJM.org).

For the usability assessment, HCWs 18 years or older involved in routine TB care at clinical sites in South Africa and India were purposively sampled (Supplementary Methods). HCWs included clinicians (e.g., medical doctors, clinical officers), laboratory technicians, and nurses.

### Study Procedures:

Demographic and clinical data were collected from participants who provided informed consent and managed using Research Electronic Data Capture (REDCap) software hosted by the University of California San Francisco.^4,5^ Blood was collected for HIV testing and diabetes screening, and tongue swabs plus up to three spot sputum samples were collected for TB testing. For participants unable to expectorate spontaneously, sputum induction was performed. Tongue swabs were collected prior to sputum per MiniDock TB test instructions (Supplementary Methods).

Usability was assessed in simulated-use sessions at healthcare facilities, where participants independently performed the test using only the manufacturer-provided instructions for use (Supplementary File). Task performance, errors, and user feedback were systematically documented and analyzed; full details are in the Supplementary Methods.

### Index Test:

Index testing used design-locked, Good Manufacturing Practices (GMP)-manufactured MiniDock MTB components (Table S2). Testing followed the manufacturer’s instructions (Supplementary File) prior to reference or comparator testing and typically within 24 hours of sample collection, except for one tongue swab and eight sputum samples processed 2–3 days later. Results were interpreted visually using MiniDock Ultra indicator lights and verified with Pluslife software. Results were classified as negative, positive, or invalid/error, with one repeat test allowed for invalid/error results.

### Comparator Tests:

LED fluorescence microscopy was performed on smears of decontaminated sputum following WHO-recommended protocols.^6^ Ultra was performed on the remaining portion of the first sputum sample (Supplementary Methods). If the initial Ultra result was invalid/error or yielded a “Trace” semiquantitative result, testing was repeated.

### Reference Standard:

The primary reference standard was the microbiological reference standard, based on mycobacterial culture using liquid mycobacterial growth indicator tube (MGIT) media, per the WHO Technical Specifications Series (TSS)-17.^7^ The secondary reference standard was the composite reference standard (Supplementary Methods). Participants were classified as TB-positive, TB-negative, or indeterminate. Operators performing molecular and reference tests were blinded to other test results.

### Statistical Analysis:

Assuming 20% TB prevalence, a target sample size of 1000 participants was set to ensure sufficient precision in sensitivity and specificity estimates, which were calculated against the microbiological reference standard, with their exact binomial 95% confidence intervals (CIs), both overall and within key subgroups. Confidence intervals were not adjusted for multiplicity. Differences between the index and comparator tests were assessed using McNemar’s test for paired proportions. The primary analysis used the first index test result. Invalid results were excluded but reported separately (Supplementary Methods).

For the usability assessment, the System Usability Scale score was calculated as described previously.^8^ Descriptive statistics and thematic analysis were used to summarize participant characteristics, survey responses, and qualitative data (Supplementary Methods).

### Ethics Statement:

The study was registered with ClinicalTrials.gov, NCT04923958.^9^ Ethical approval was obtained from institutional review boards and/or research ethics committees at all participating institutions (Table S1). Written informed consent was obtained from all participants or caregivers, along with verbal assent from adolescents. Participation did not affect the standard of care provided.

This study adheres to established guidelines for reporting diagnostic accuracy studies.^10^

## Results

### Study Population:

**Figure 1:**
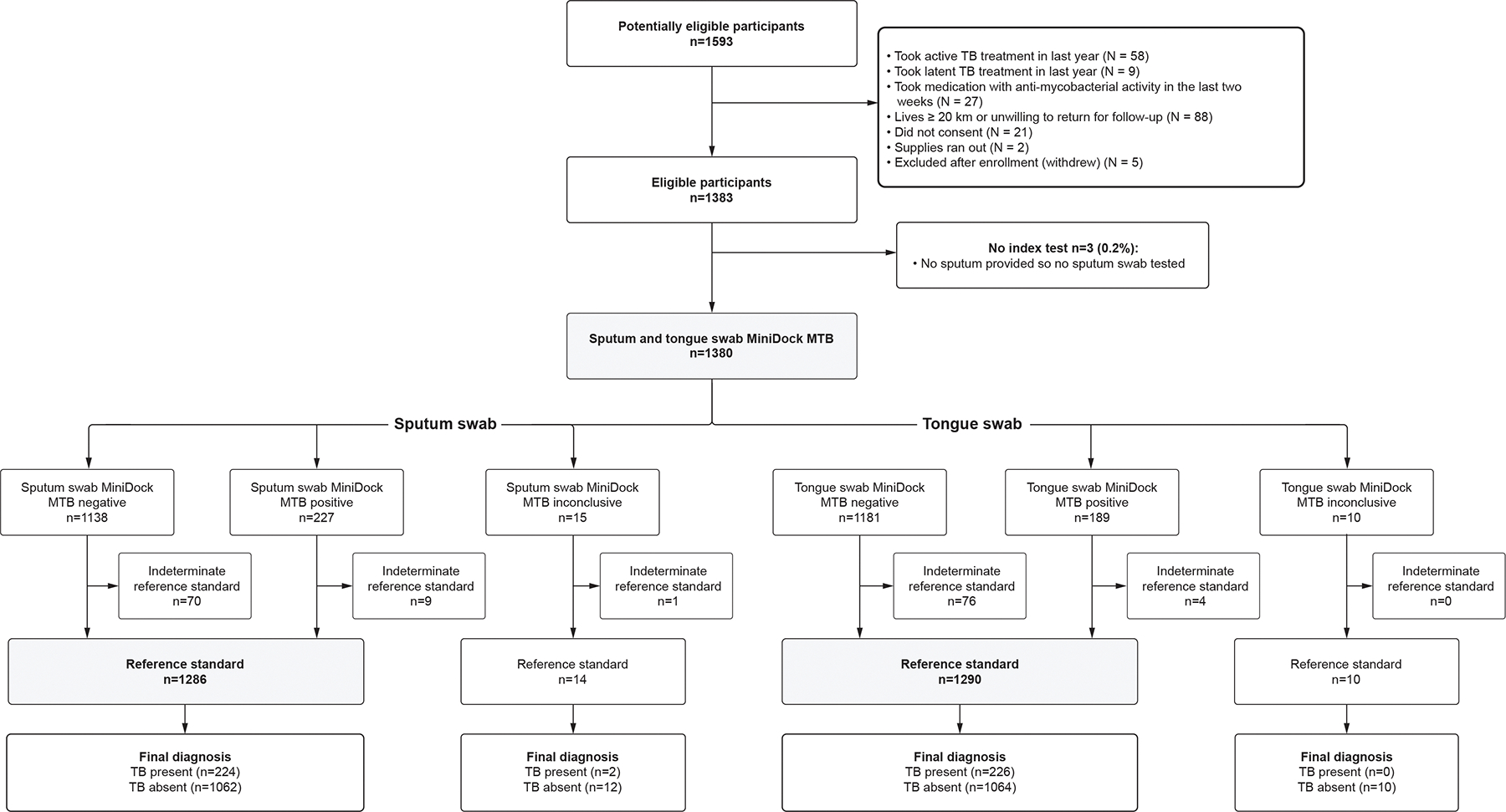
Participant Flow diagram

Of 1593 adults and adolescents screened, 210 were excluded for reasons outlined in Figure 1. Of the 1,383 participants enrolled, 1,380 provided samples and were included in the analysis.

Among these, 603 (43.7%) were female, and the median age was 41 years (interquartile range, 29–54) (Tables 1 and S3). HIV infection was reported in 255 (18.6%), and 187 (13.6%) had diabetes. Based on the microbiological reference standard, 226 (17.4%) were diagnosed with confirmed TB, all referred for treatment according to local guidelines.

Bacillary load as measured by Ultra semi-quantitative grade was higher at the four African sites compared to the three Asian sites (56.7–83.3% vs. 38.9–54.5% of Xpert Ultra-positive participants with medium or high grades). TB prevalence varied by country, ranging from 4.8% in India to 28.1% in Nigeria. Sputum induction was required in 144 (10.4%) participants.

### Performance of MiniDock MTB on Sputum:

Among sputum MiniDock MTB tests conducted, 15 (1.1%) produced invalid results initially (Table S4). Retesting residual lysate yielded valid results in 10 cases (67%). After exclusions for missing index or reference test results, 1286 (93.2%) participants were included in the analysis.

**Figure 2:**
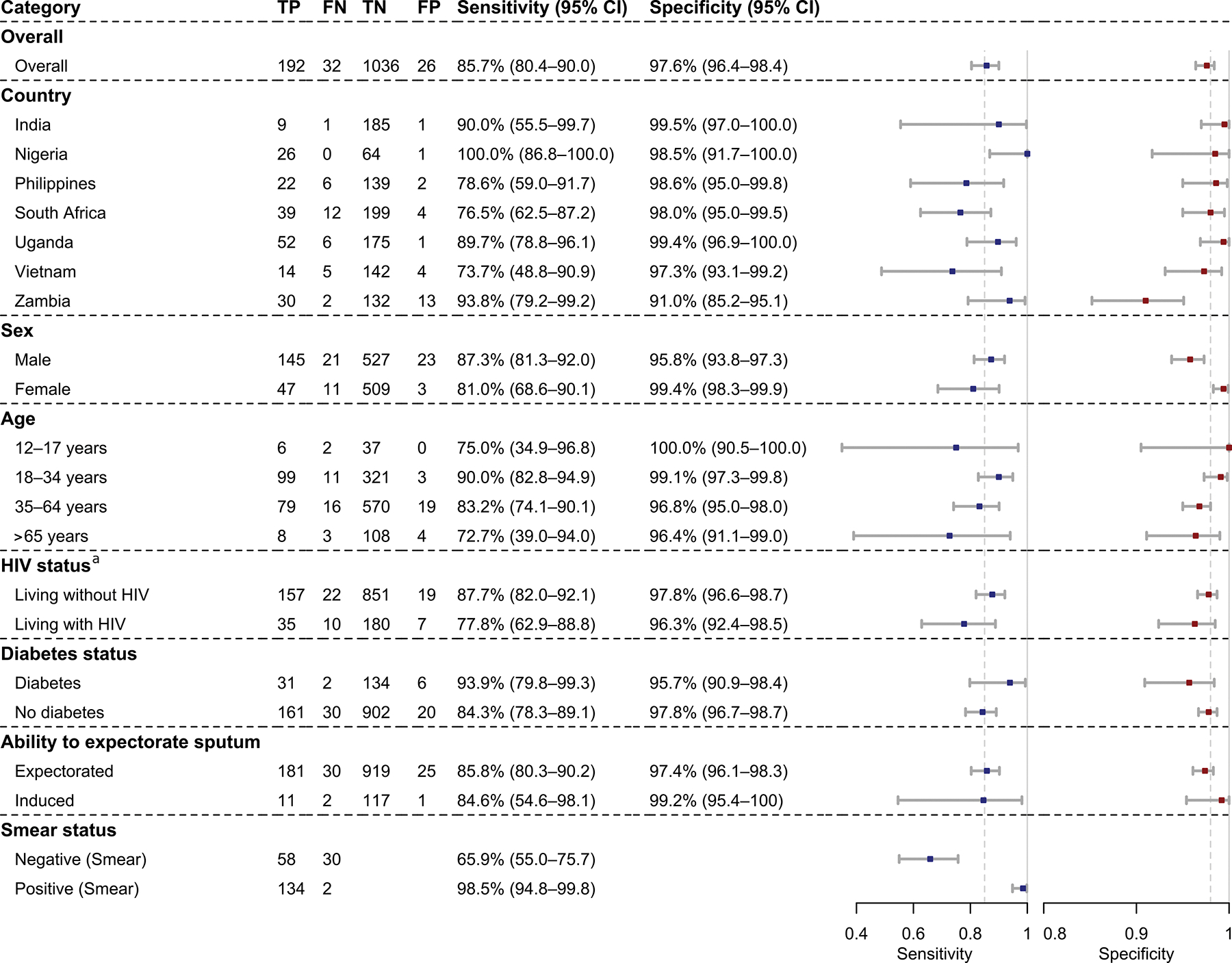

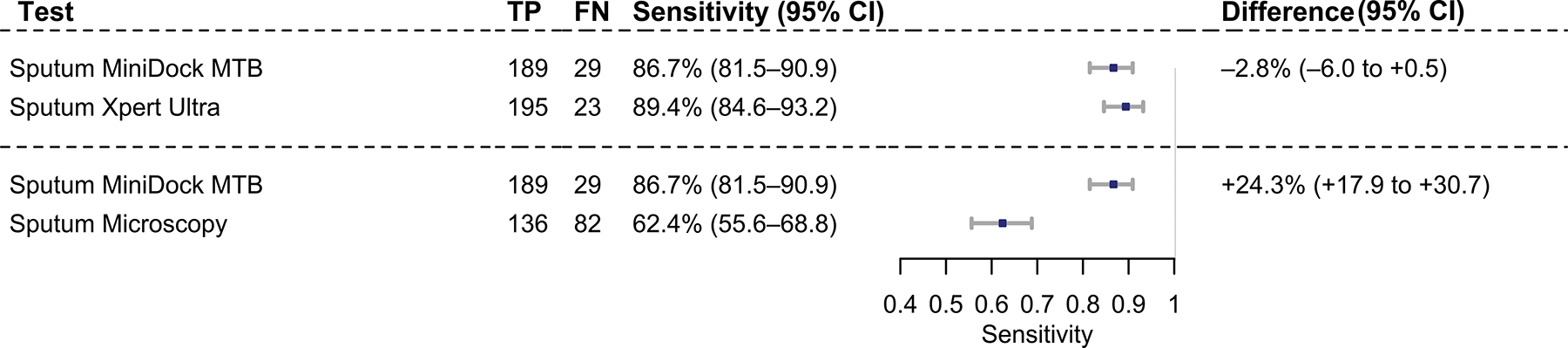

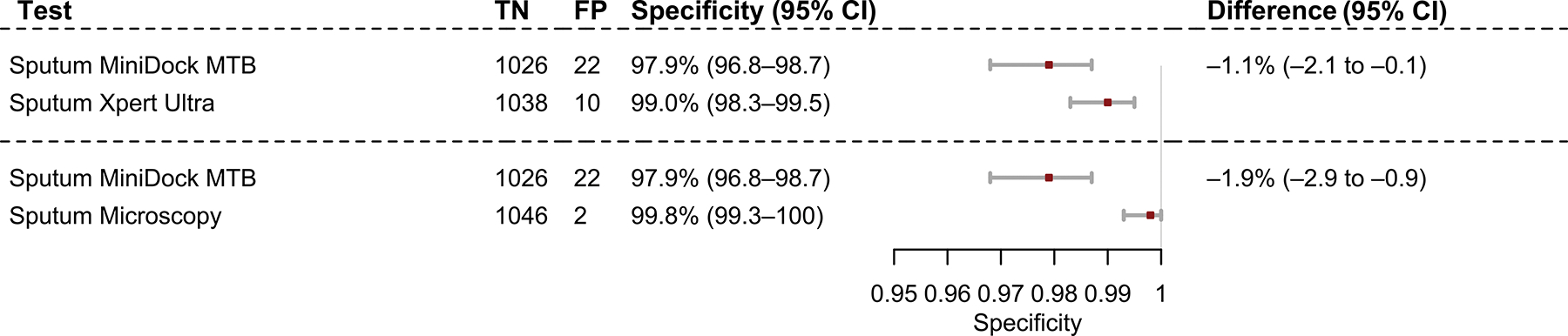
Performance of sputum MiniDock MTB against MRS. **(A)** Diagnostic accuracy overall and by subgroup. The dashed line represents the WHO TPP performance targets for a sputum-based near-POC TB test (sensitivity ≥85% and specificity ≥98%). **(B)** Head-to-head sensitivity comparison with conventional sputum-based tests. **(C)** Head-to-head specificity comparison with other sputum-based TB tests. Abbreviations: TP, True Positive; FN, False Negative; TN, True Negative; FP, False Positive; CI, Confidence Interval; HIV: Human Immunodeficiency Virus.

Sputum MiniDock MTB demonstrated a sensitivity of 85.7% (95% CI, 80.4–90.0) and specificity of 97.6% (95% CI, 96.4–98.4) (unstratified, Figure 2A). Sensitivity varied across countries, ranging from 73.7% to 100%. Specificity was high in all countries except Zambia (91.0% [95% CI, 85.2–95.1]). Sensitivity was slightly higher among males than females (87.3% [95% CI, 81.3–92.0] v. 81.0% [95% CI, 68.6–90.1]) and in people without HIV compared to those with HIV (87.7% [95% CI, 82.0–92.1] v. 77.8% [95% CI, 62.9–88.8]). Performance was similar between participants able to spontaneously expectorate and those requiring sputum induction. Sensitivity among smear-positive individuals was 98.5% (95% CI, 94.8–99.8), compared to 65.9% (95% CI, 55.0–75.7) among smear-negative, TB-positive ones. Including repeat test results did not change overall sensitivity or specificity (Table S5). In the secondary analysis using the composite reference standard, unstratified sensitivity was lower and specificity higher (Table S6).

Sputum MiniDock MTB showed sensitivity comparable to sputum Ultra (86.7% vs. 89.4%; difference −2.8% [95% CI: −6.0–0.5]) and higher than smear microscopy (86.7% vs. 62.4%; difference 24.3% [95% CI: 17.9–30.7]) (Figure 2B). Specificity was lower than that of Ultra (97.9% vs. 99.8%; difference −1.9% [95% CI: −2.9-−0.9]) (Figure 2C). Analyses using composite reference standard yielded a larger sensitivity difference with Ultra (overlapping CIs) (Table S7). Treating Ultra trace results as positive reduced the specificity difference but increase the sensitivity difference (Table S8).

Positive concordance with sputum Ultra was high across all semi-quantitative categories, except very low and trace, where it was lower (Table S9).

### Performance of MiniDock MTB on Tongue Swabs:

Of the MiniDock MTB tests performed on tongue swabs, 10 (0.8%) initially yielded invalid results (Table S4). All were successfully resolved through repeat testing (Table S4). After exclusions for missing index or reference test results, 1290 (93.5%) participants were included in the analysis.

**Figure 3:**
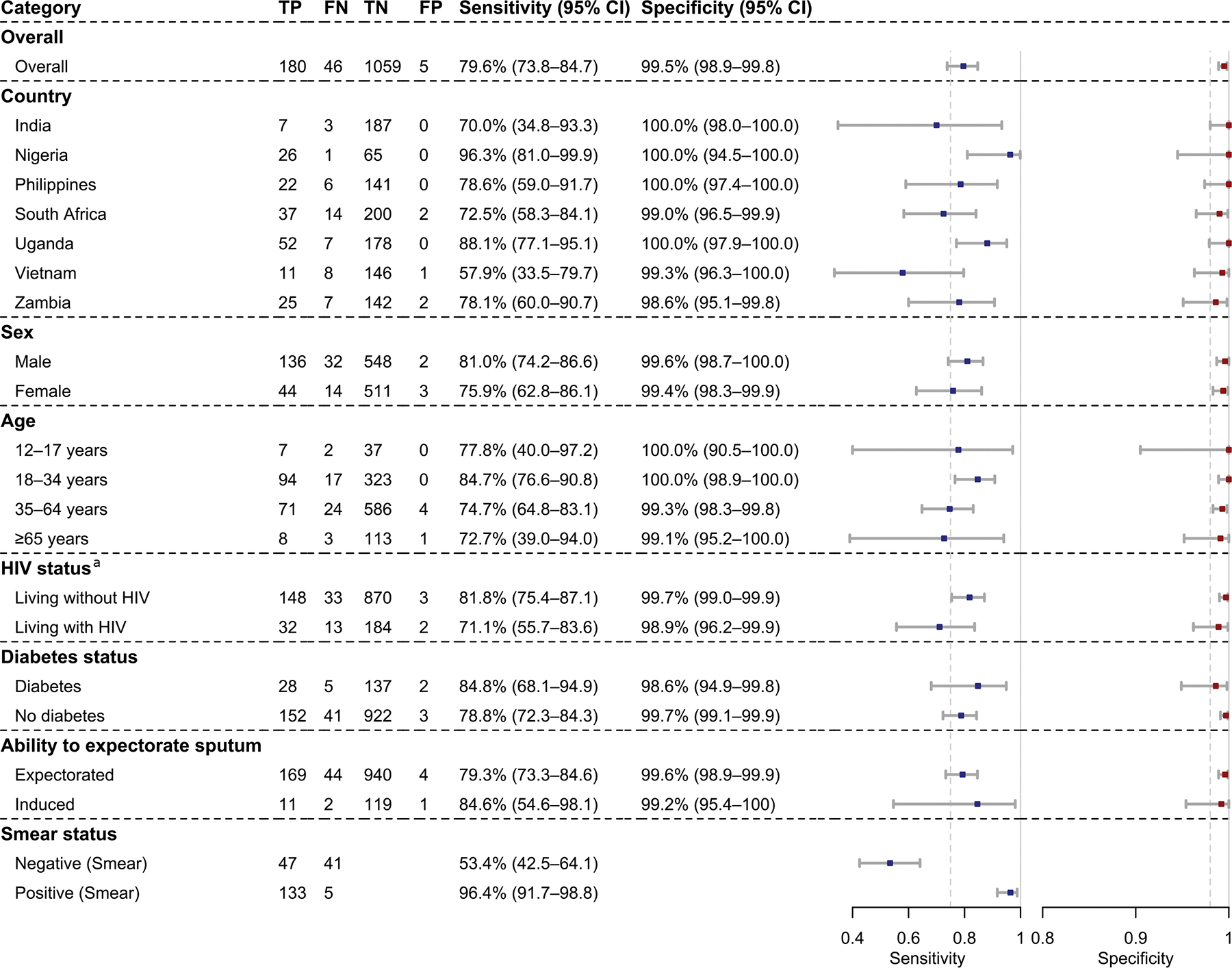

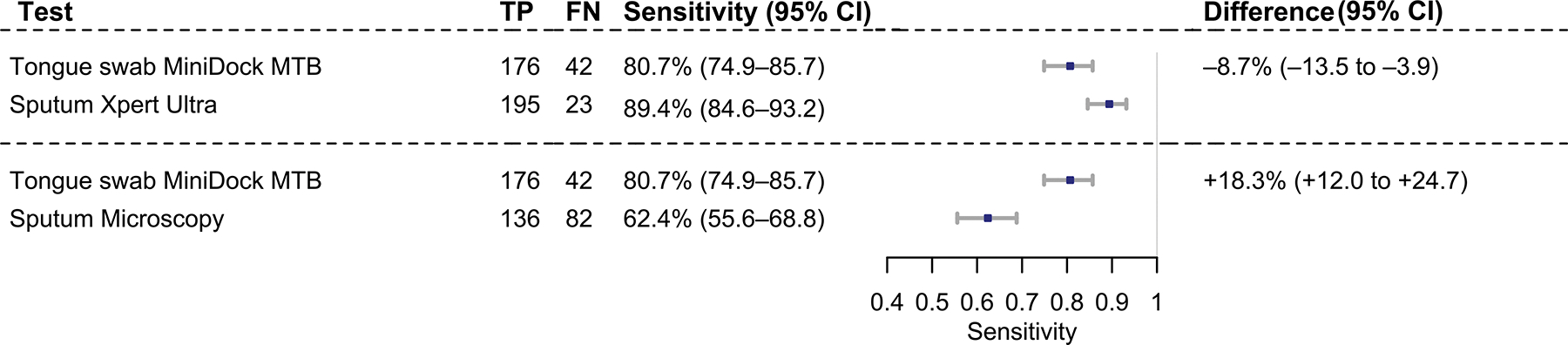

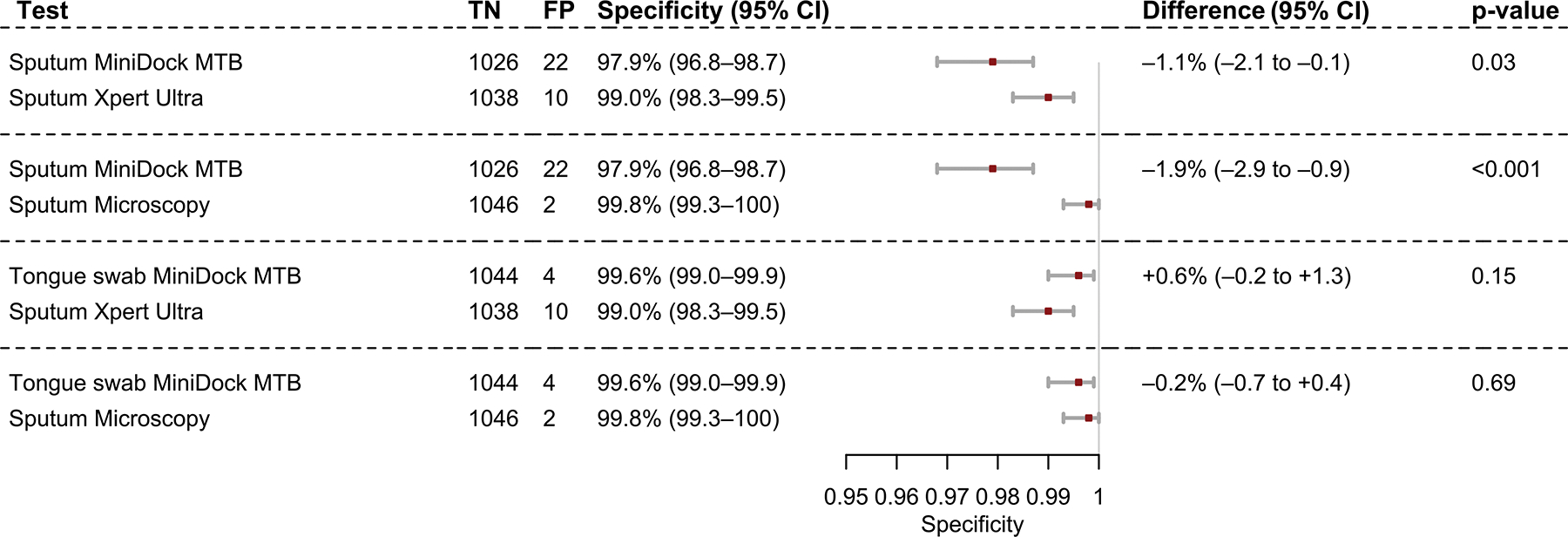
Performance of tongue swab MiniDock MTB against MRS. **(A)** Diagnostic accuracy overall and by subgroup. The dashed line represents the WHO TPP performance targets for a non-sputum-based near-POC TB test (sensitivity ≥75% and specificity ≥98%). **(B)** Head-to-head sensitivity comparison with conventional sputum-based tests. **(C)** Head-to-head specificity comparison with other sputum-based TB tests. Abbreviations: TP, True Positive; FN, False Negative; TN, True Negative; FP, False Positive; CI, Confidence Interval; HIV: Human Immunodeficiency Virus.

Tongue swab MiniDock MTB showed a sensitivity of 79.6% (95% CI, 73.8–84.7) and specificity of 99.5% (95% CI, 98.9–99.8) (unstratified, Figure 3A). Sensitivity varied by country from 57.9% to 96.3%, while specificity remained consistently high. Sensitivity was slightly higher in males than females (81.0% [95% CI, 74.2–86.6] v. 75.9% [95% CI, 62.8–86.1]), and in people without HIV compared to those with HIV (81.8% [95% CI, 75.4–87.1] v. 71.1% [95% CI, 55.7–83.6]). Among smear-positive cases, sensitivity reached 96.4% (95% CI, 91.7–98.8), dropping to 53.4% (95% CI, 42.5–64.1) in smear-negative cases. For participants requiring sputum induction, sensitivity was 84.6% (95% CI, 54.6–98.1) and specificity 99.2% (95% CI, 95.4–100). Including repeat test results did not materially alter sensitivity or specificity (Table S5). When the composite reference standard was used, overall sensitivity decreased (Table S6).

Tongue swab MiniDock MTB sensitivity exceeded sputum smear microscopy (80.7% vs. 62.4%; difference 18.3% [95% CI: 12.0–24.7]) but was lower than sputum Ultra (80.7% vs. 89.4%; difference −8.7% [95% CI: −13.5-−3.9]) (Figure 3B). Specificity was comparable to both smear microscopy and Ultra (Figure 3C). Composite reference standard analyses showed greater sensitivity differences with Ultra but smaller with microscopy (Tables S7). Including trace results as positive increased the differences (Tables S8).

Concordance with sputum Ultra was high for high and medium semi-quantitative categories, but declined progressively in low, very low, and trace categories (Table S10).

When sputum and tongue swab results were combined, sensitivity and specificity were 87.6% (95% CI, 82.6–91.6) and 97.2% (95% CI, 96.0–98.1), respectively (Table S11). No adverse events related to MiniDock MTB were observed.

### Usability Results:

**Figure 4:**
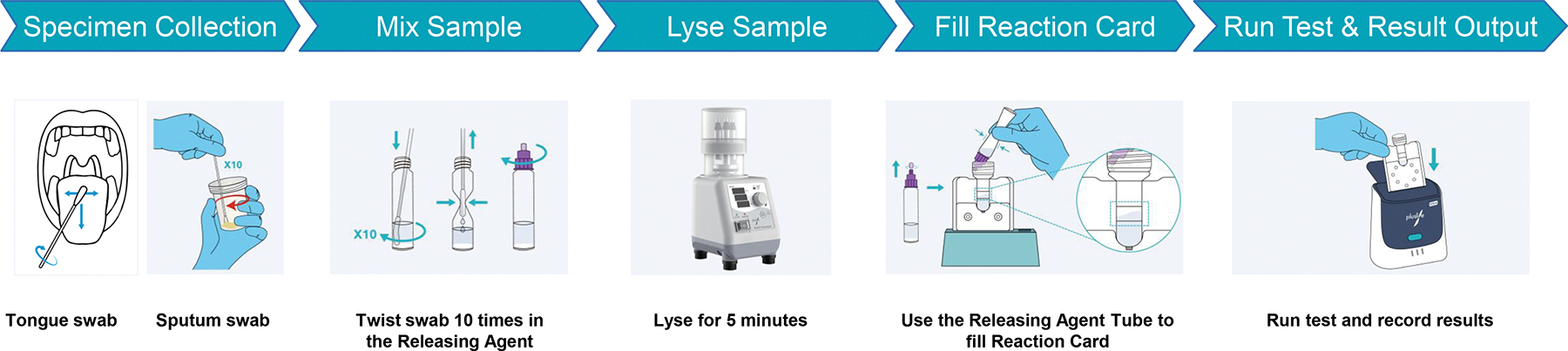

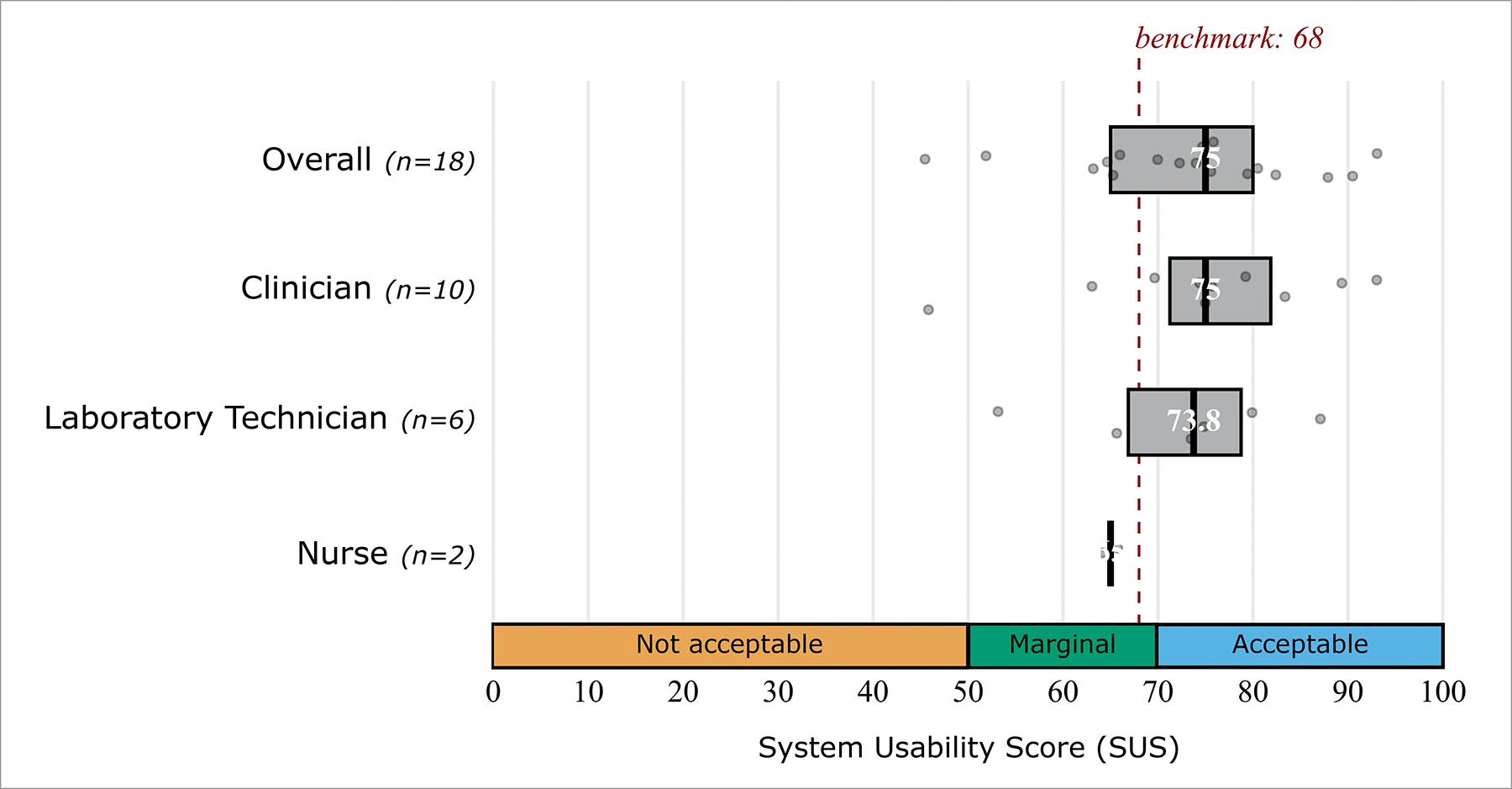
Usability of MiniDock MTB. **(A)** Overview of the sequential steps from sample collection to result interpretation in the MiniDock MTB Test workflow. **(B)** System Usability Score (SUS) overall and per occupation; higher scores indicate better usability.

Eighteen HCWs participated in the usability evaluation (10 from India and 8 from South Africa). The median age was 29 years (IQR 28–33), with a median of 6 years (IQR 3–10) of professional experience and 3 years (IQR 1–5) in TB care (Table S12). All were test-naïve and relied solely on the instructions for use (Figure 4A, Supplementary File).

The MiniDock MTB Test received a median System Usability Scale score of 75 (IQR 65–80) (Figure 2B, Figure S1), consistent within the “acceptable” usability range. Scores varied by user group: clinicians reported the highest ratings (median 75, IQR 70–82.5), followed by laboratory technicians (median 73.8, IQR 65–80), while nurses reported the lowest (65 for both participants) (Figure 2B).

Most core procedural steps (>90%) were completed successfully (Table S13). However, among the 22 tasks assessed, two critical errors were identified: omission of the lysis step and overfilling the reaction card (Table S14).

Most users found the test acceptable (n=17/18) (Figure S2). In open-ended feedback, the test was consistently described as easy-to-use, especially compared to sputum smear microscopy (Table S15). Users also valued its compatibility with non-sputum samples and potential for decentralized use.

## Discussion

In this multi-country evaluation, the MiniDock MTB test demonstrated diagnostic accuracy consistent with WHO target product profile for near-POC TB diagnostics using sputum and tongue swab specimens.^3^ In addition to robust performance, the test was rated as easy-to-use, with high usability scores indicating that it can be operated with minimal training. These findings support the feasibility of implementing MiniDock MTB as a flexible, user-friendly diagnostic option that performs comparably to or better than established TB diagnostic tests while offering the opportunity for noninvasive sampling and supporting universal access to molecular TB testing.

Earlier studies of swab-based molecular TB testing evaluated tongue swabs processed with laboratory-based in-house qPCR assays or adapted sputum tests like Ultra, demonstrating their feasibility as a noninvasive option, though sensitivity varied by bacillary load and assay type.^11–20,19^ However, most approaches adapted tests optimized for large volume (0.5–2 mL) sputum samples rather than being purpose-built for swabs. In contrast, MiniDock MTB was designed for swab-based TB detection. Its development incorporated insights from earlier work, including the need to enhance MTB lysis using heat and mechanical treatment, and to eliminate complex sample preparation steps through an extraction-free workflow^18,21^

While this study design addressed key limitations of prior assays, performance still varied across settings and populations for both sputum and tongue swab, likely reflecting differences in disease severity, timing of healthcare-seeking behavior, underlying bacterial load, and the prevalence of low-IS*6110*-copy strains.^22–26^ Consistent with this, lower sensitivity was observed among individuals with presumed low mycobacterial burden, such as people living with HIV, those with smear-negative TB, or with lower Ultra semi-quantitative grades, though to varying degrees. This finding aligns with previously reported Ultra performance in similar subpopulations,^27–29^ further underscoring the challenges of TB diagnosis in paucibacillary disease contexts.

Despite these challenges, our findings demonstrate that MiniDock MTB offers reasonable accuracy with practical advantages. Our accuracy findings, combined with the test’s ease of use and anticipated low cost (<$180 USD per device and <$4 USD per test), have implications for the future of TB diagnostics. MiniDock MTB has the potential to be a tool enabling increased access to molecular testing, including for the 11–43% of adults unable to produce sputum, and by increasing diagnostic yield.^30^ Its usability and acceptability among test-naïve users relying solely on the manufacturer-provided instructions for use underscore its suitability for decentralized use with minimal training. Furthermore, the test meets many WHO target product profile criteria for a near-POC test: it is low-cost, rapid, compact, simple to use, extraction-free, and well suited for decentralized or resource-limited settings.^3^ As such, it offers a feasible, alternative to microscopy that could expand access to molecular TB diagnosis, improve case detection, and bring high-quality testing closer to where patients live and seek care.

The study findings supported WHO guideline development group review of MiniDock MTB as a near-POC test for TB. Provided the recommendations are positive, implementing MiniDock MTB aligns directly with WHO’s End TB Strategy, which emphasizes early, equitable access to high-quality diagnosis, and WHO guidelines that recommend replacing smear microscopy with WHO-recommended rapid molecular diagnostics at all health system levels, including primary care.^31,32^

This study has several notable strengths. The multi-center design across diverse geographic and clinical contexts enhances generalizability, while the prospective, cross-sectional methodology and standardized protocols support internal validity. The inclusion of a usability assessment further strengthens the study by evaluating operational feasibility and end-user interaction, critical components for widespread implementation in resource-limited settings. However, the study also has limitations. The study was conducted in research settings under controlled conditions, potentially inflating accuracy estimates. The use of an imperfect reference standard, particularly in HIV-positive or paucibacillary subgroups, could have affected specificity estimates. Further, while the overall sample size was sufficient for primary analyses, some subgroups may have been underrepresented, limiting the precision of subgroup-specific estimates.

Although results exceeded accuracy targets for near-POC TB tests, several implementation questions remain to be addressed by future studies. First, we observed that sensitivity decreased with lower mycobacterial load, particularly for tongue swabs. This pattern aligns with previous studies and highlights a potential limitation of tongue swab-based sampling for detecting paucibacillary disease.^18,21^ The applicability of tongue swab testing in populations with low bacillary burden, such as individuals undergoing active case finding in community-based screening programs or children, remains unclear and warrants dedicated evaluation. Moreover, studies are needed to assess diagnostic yield, rather than accuracy alone, especially in populations likely to have paucibacillary disease, and to evaluate real-world patient-relevant outcomes such as turnaround time. Second, the current MiniDock platform does not support drug-resistance testing, highlighting the need to develop and evaluate diagnostic algorithms that incorporate reflex testing strategies.

## Conclusions

MiniDock MTB demonstrated diagnostic accuracy and usability for TB detection using sputum and tongue swab samples across diverse settings. Its near-POC design and minimal training requirements could expand access to timely diagnosis, particularly in peripheral health centers in high burden settings that continue to rely on smear microscopy or do not currently offer TB testing.

## Supplementary Material

supplement

## Figures and Tables

**Table 1:** Demographic and clinical characteristics of study participants.*

	All	IN	NG	PH	SA	UG	VN	ZM
**Total Enrolled,** *N (%)*	1380 (100%)	210 (15.2%)	96 (7.0%)	182 (13.2%)	270 (19.6%)	262 (19.0%)	180 (13.0%)	180 (13.0%)
**Female,** *N (%)*	603 (43.7%)	91 (43.3%)	41 (42.7%)	91 (50.0%)	137 (50.7%)	102 (38.9%)	81 (45.0%)	60 (33.3%)
**Age,** *median (IQR)*	41 (29–54)	51 (36–61)	35 (26–45)	50 (33–61)	37 (28–46)	34 (23–45)	53 (40–64)	37 (29–46)
**Symptomatic** ^a^, *N (%)*	1342 (97.2%)	207 (98.6%)	96 (100%)	179 (98.4%)	258 (95.6%)	243 (92.7%)	180 (100%)	179 (99.4%)
**Cough ≥ 2 weeks,** *N (%)*	1313 (95.1%)	207 (98.6%)	96 (100%)	175 (96.2%)	247 (91.5%)	236 (90.1%)	180 (100%)	172 (95.6%)
**Previous history of TB,** *N (%)*	236 (17.1%)	19 (9.0%)	6 (6.3%)	42 (23.1%)	69 (25.6%)	26 (9.9%)	38 (21.1%)	36 (20.0%)
**HIV status positive** ^b^, *N (%)*	255 (18.5%)	1 (0.5%)	6 (6.3%)	2 (1.1%)	96 (35.6%)	76 (29.0%)	5 (2.8%)	69 (38.3%)
**Diabetes status positive** ^c^, *N (%)*	187 (13.6%)	57 (27.1%)	6 (6.3%)	23 (12.6%)	27 (10.0%)	33 (12.6%)	18 (10.0%)	23 (12.8%)
**Sputum smear status positive,** *N (%)*	144 (10.4%)	6 (2.9%)	29 (30.2%)	19 (10.4%)	18 (6.7%)	47 (17.9%)	7 (3.9%)	18 (10.0%)
**Sputum Ultra positive** ^d^, *N (%)*	213 (15.7%)	11 (5.2%)	30 (31.6%)	28 (15.4%)	41 (15.6%)	55 (21.2%)	18 (10.6%)	30 (16.8%)
**MRS status positive,** *N (%)*	226 (16.4%)	10 (4.8%)	27 (28.1%)	28 (15.4%)	51 (18.9%)	59 (22.5%)	19 (10.6%)	32 (17.8%)

Abbreviations: IN: India; NG: Nigeria; PH: Philippines; SA: South Africa; UG: Uganda; VN: Viet Nam; ZA: Zambia; IQR: inter-quartile range; TB: tuberculosis; MRS: microbiological reference standard

a Symptomatic is defined as any one of the following symptoms: cough ≥ 2 weeks, fever, weight loss, night sweats

b HIV positivity defined by self-reported positivity or positive HIV test at baseline.

c Diabetes defined by self-reported history of diabetes, currently taking diabetes medications or insulin, or an hba1c result >=6.5%.

d Ultra considered positive with semi-quantification result of very low or higher.
