# Supplementary material for: Pulmonary Tuberculosis Detection with MiniDock MTB Using Swab Samples
Source: N Engl J Med. Author manuscript; Available in PMC 2026 Apr 30. (PMC13132025; doi:10.1056/NEJMoa2509761)
Supplement: supplement [file NIHMS2134962-supplement-supplement.pdf]

# Table of Contents

**Consortium Members** ... 3
**Supplementary Methods** ... 11
Study Objectives ... 11
Eligibility Criteria ... 11
Study Procedures ... 11
Index Test ... 12
Comparator Tests ... 12
Reference Standard ... 13
Outcome Measures ... 13
Sample Size Estimation ... 13
Data Analysis ... 13
STARD Reporting Checklist ... 14
**Supplementary Figures** ... 18
**Figure S1:** Distribution of responses to individual system usability scale (SUS) items regarding the use of MiniDock MTB among health care workers in India (n=10) and South Africa (n=8). ... 18
**Figure S2:** Acceptability of MiniDock MTB ... 19
**Supplementary Tables** ... 20
**Table S1:** Enrollment sites and ethics committees* ... 20
**Table S2:** Kit lot numbers and instrument serial numbers of the Guangzhou Pluslife Biotech equipment and reagents per country. ... 21
**Table S3:** Demographic and clinical characteristics of study participants* ... 22
**Table S4:** MiniDock MTB invalid rates, overall and by country* ... 24
**Table S5:** Diagnostic accuracy compared to MRS, including repeat test results ... 25
**Table S6:** Diagnostic accuracy compared to CRS* ... 26
**Table S7:** Head-to-head diagnostic accuracy comparison between MiniDock MTB and conventional sputum-based TB tests against CRS* ... 27
**Table S8:** Sensitivity analysis of the head-to-head diagnostic accuracy comparison between MiniDock MTB and sputum Xpert Ultra against MRS, including Ultra trace results ... 28
**Table S9:** Concordance between sputum MiniDock MTB and sputum Xpert Ultra test results, overall and by Xpert Ultra semi-quantitative grade. ... 29
**Table S10:** Concordance between tongue swab MiniDock MTB and sputum Xpert Ultra test results, overall and by Xpert Ultra semi-quantitative grade. ... 30
**Table S11:** Diagnostic accuracy compared to MRS of combined tongue swab and sputum MiniDock MTB test results* ... 31
**Table S12:** Usability study participant characteristics ... 32
**Table S13:** Summary of task completion and observed error rates during usability evaluation of MiniDock MTB ... 33
**Table S14:** Summary of use errors during MiniDock MTB testing and the potential risks associated with the errors. ... 34

**Table S15:** Summary of qualitative findings on usability and preliminary acceptability of MiniDock MTB....................................................................................................................35
**References**....................................................................................................................37

## Consortium Members

| First name | Surname | Affiliation |
|---|---|---|
| **India** | | |
| Devasahayam J. | Christopher | Christian Medical College, Vellore |
| Shanmugasundaram | Elango | Christian Medical College, Vellore |
| Jerusha | Emmanuel | Christian Medical College, Vellore |
| Vinita | Ernest | Christian Medical College, Vellore |
| Priyadarshini | Gajendran | Christian Medical College, Vellore |
| Flavita | John | Christian Medical College, Vellore |
| Bharath | Karthikeyan | Christian Medical College, Vellore |
| Divya | Mangal | Christian Medical College, Vellore |
| Swetha | Sankar | Christian Medical College, Vellore |
| Rajasekar | Sekar | Christian Medical College, Vellore |
| Reena | Sekar | Christian Medical College, Vellore |
| Deepa | Shankar | Christian Medical College, Vellore |
| Mary | Shibiya | Christian Medical College, Vellore |
| Balamugesh | Thangakunam | Christian Medical College, Vellore |
| Sai | Vijayasree | Christian Medical College, Vellore |
| **Philippines** | | |
| Jared | Almonte | De La Salle Medical and Health Sciences Institute, Cavite |
| Kevin Joshua | Alonzo | National TB Reference Laboratory, Research Institute for Tropical Medicine, Department of Health |
| Mary Faith | Angcaya | De La Salle Medical and Health Sciences Institute, Cavite |
| Joseph Edwin L. | Bascuña | National TB Reference Laboratory, Research Institute for Tropical Medicine, Department of Health |
| Ramon P. | Basilio | National TB Reference Laboratory, Research Institute for Tropical Medicine, Department of Health |
| Asella Ruvijean | Cariaga | De La Salle Medical and Health Sciences Institute, Cavite |
| Gabriella | Castillon | De La Salle Medical and Health Sciences Institute, Cavite |
| Victoria | Dalay | De La Salle Medical and Health Sciences Institute, Cavite |
| Karlo | Dayawon | De La Salle Medical and Health Sciences Institute, Cavite |
| Raul | Destura | National Institutes of Health, University of the Philippines Manila |
| Jezreel | Esguerra | De La Salle Medical and Health Sciences Institute, Cavite |
| Eleonor | Garcia | De La Salle Medical and Health Sciences Institute, Cavite |
| Darecil | Gelina | De La Salle Medical and Health Sciences Institute, Cavite |

| First name | Surname | Affiliation |
|---|---|---|
| Joseph Aldwin | Goleña | De La Salle Medical and Health Sciences Institute, Cavite |
| Maria Marissa | Golla | De La Salle Medical and Health Sciences Institute, Cavite |
| Emmanuelle | Gutierrez | De La Salle Medical and Health Sciences Institute, Cavite |
| Gidalthi Jonathan | Ilagan | De La Salle Medical and Health Sciences Institute, Cavite |
| Dodge R. | Lim | National TB Reference Laboratory, Research Institute for Tropical Medicine, Department of Health |
| Jaiem | Maranan | De La Salle Medical and Health Sciences Institute, Cavite |
| Danaida | Marcelo | De La Salle Medical and Health Sciences Institute, Cavite |
| Leonedy | Masangcay | De La Salle Medical and Health Sciences Institute, Cavite |
| Jenkin | Mendoza | National TB Reference Laboratory, Research Institute for Tropical Medicine, Department of Health |
| Angelita | Pabruada | De La Salle Medical and Health Sciences Institute, Cavite |
| Laarean | Perlas | De La Salle Medical and Health Sciences Institute, Cavite |
| Annalyn | Reyes | De La Salle Medical and Health Sciences Institute, Cavite |
| Roeus Vincent Arjay G. | Reyes | National TB Reference Laboratory, Research Institute for Tropical Medicine, Department of Health |
| Lorenzo | Reyes | National TB Reference Laboratory, Research Institute for Tropical Medicine, Department of Health |
| Maria Guileane | Sanchez-Pogosa | National TB Reference Laboratory, Research Institute for Tropical Medicine, Department of Health |
| Maricef | Tonquin | De La Salle Medical and Health Sciences Institute, Cavite |
| Charles | Yu | De La Salle Medical and Health Sciences Institute, Cavite |
| **Netherlands** | | |
| Kristin | Kremer | KNCV Tuberculosis Foundation, The Hague |
| Petra | Da Haas | KNCV Tuberculosis Foundation, The Hague |
| Bianca | Tasca | KNCV Tuberculosis Foundation, The Hague |
| **Nigeria** | | |
| John Samson | Bimba | Zankli Research Center, Bingham University, Karu |
| Chukwuma | Anyaike | Federal Ministry of Health and Social Welfare, Abuja |
| Ngozi Juliana | Ebisike | Federal Capital Territory Department of Health and Human Services, Abuja |
| Bukola Adeyoola | Ajide | Zankli Research Center, Bingham University, Karu |

| First name | Surname | Affiliation |
|---|---|---|
| Paul Bature | Ndaks | Zankli Research Center, Bingham University, Karu |
| Kushim Jonathan | Agwom | Zankli Research Center, Bingham University, Karu |
| Emmana Ekpenyong | Bassey | Zankli Research Center, Bingham University, Karu |
| Timan Eliya Taryuta | Eliya | Zankli Research Center, Bingham University, Karu |
| Precious Hauwa | Ishaku | Zankli Research Center, Bingham University, Karu |
| Paul Bassi | Amos | Zankli Research Center, Bingham University, Karu |
| Abigail Abi | Daniel | Zankli Research Center, Bingham University, Karu |
| Asmau Adelodun | Bashir | Zankli Research Center, Bingham University, Karu |
| Sarah | Asugu | Zankli Research Center, Bingham University, Karu |
| Godwin Chikezie | Okorie | Zankli Research Center, Bingham University, Karu |
| Ndubuisi Innocent | Odanwu | Township Primary Health Care Gwagwalada, Abuja |
| Ndubisi Stephen | Oko | Township Primary Health Care Gwagwalada, Abuja |
| Suleiman | Ibrahim | Township Primary Health Care Gwagwalada, Abuja |
| Abubakar | Adamu | Township Primary Health Care Gwagwalada, Abuja |
| Mohammed Buhari | Alhassan | Township Primary Health Care Gwagwalada, Abuja |
| Iliyasu Ozovehe | Yusuf | Township Primary Health Care Gwagwalada, Abuja |
| Christie Chinyere | Dick | Nyanya General Hospital, Abuja |
| Bernerd Tersur | Adean | Nyanya General Hospital, Abuja |
| Alfa Johnson | Alabi | Nyanya General Hospital, Abuja |
| Tosin Abigail | Jaiyeola | Nyanya General Hospital, Abuja |
| Tamar Helfiyada | Joseph | Nyanya General Hospital, Abuja |
| Josiah | Ocheni | Nyanya General Hospital, Abuja |
| **South Africa** | | |
| Shima | Abdulgadar | DSI-NRF Centre of Excellence for Biomedical Tuberculosis Research, South African Medical Research Council Centre for Tuberculosis Research, Division of Molecular Biology and Human Genetics, Faculty of Medicine and Health Sciences, Stellenbosch University, Cape Town |
| Cammy | Botha | DSI-NRF Centre of Excellence for Biomedical Tuberculosis Research, South African Medical Research Council Centre for Tuberculosis Research, Division of Molecular Biology and Human Genetics, Faculty of Medicine and Health Sciences, Stellenbosch University, Cape Town |
| Brigitta | Derendinger | DSI-NRF Centre of Excellence for Biomedical Tuberculosis Research, South African Medical Research Council Centre for Tuberculosis Research, Division of Molecular Biology and Human Genetics, Faculty of Medicine and Health Sciences, Stellenbosch University, Cape Town |
| Welile | Dube-Nwamba | DSI-NRF Centre of Excellence for Biomedical Tuberculosis Research, South African Medical Research Council Centre for Tuberculosis |

| First name | Surname | Affiliation |
|---|---|---|
| | | Research, Division of Molecular Biology and Human Genetics, Faculty of Medicine and Health Sciences, Stellenbosch University, Cape Town |
| Jane | Fortuin | DSI-NRF Centre of Excellence for Biomedical Tuberculosis Research, South African Medical Research Council Centre for Tuberculosis Research, Division of Molecular Biology and Human Genetics, Faculty of Medicine and Health Sciences, Stellenbosch University, Cape Town |
| Siphosethu | Gonya | DSI-NRF Centre of Excellence for Biomedical Tuberculosis Research, South African Medical Research Council Centre for Tuberculosis Research, Division of Molecular Biology and Human Genetics, Faculty of Medicine and Health Sciences, Stellenbosch University, Cape Town |
| Chumani | Hatile | DSI-NRF Centre of Excellence for Biomedical Tuberculosis Research, South African Medical Research Council Centre for Tuberculosis Research, Division of Molecular Biology and Human Genetics, Faculty of Medicine and Health Sciences, Stellenbosch University, Cape Town |
| Megan | Hendrikse | DSI-NRF Centre of Excellence for Biomedical Tuberculosis Research, South African Medical Research Council Centre for Tuberculosis Research, Division of Molecular Biology and Human Genetics, Faculty of Medicine and Health Sciences, Stellenbosch University, Cape Town |
| Charlotte | Lawn | DSI-NRF Centre of Excellence for Biomedical Tuberculosis Research, South African Medical Research Council Centre for Tuberculosis Research, Division of Molecular Biology and Human Genetics, Faculty of Medicine and Health Sciences, Stellenbosch University, Cape Town |
| Disha | Mathoorah | DSI-NRF Centre of Excellence for Biomedical Tuberculosis Research, South African Medical Research Council Centre for Tuberculosis Research, Division of Molecular Biology and Human Genetics, Faculty of Medicine and Health Sciences, Stellenbosch University, Cape Town |
| Desiree Lem | Mbu | DSI-NRF Centre of Excellence for Biomedical Tuberculosis Research, South African Medical Research Council Centre for Tuberculosis Research, Division of Molecular Biology and Human Genetics, Faculty of Medicine and Health Sciences, Stellenbosch University, Cape Town |
| Zintle | Ntetha | DSI-NRF Centre of Excellence for Biomedical Tuberculosis Research, South African Medical Research Council Centre for Tuberculosis Research, Division of Molecular Biology and Human Genetics, Faculty of Medicine and Health Sciences, Stellenbosch University, Cape Town |

| First name | Surname | Affiliation |
|---|---|---|
| Anna | Okunola | DSI-NRF Centre of Excellence for Biomedical Tuberculosis Research, South African Medical Research Council Centre for Tuberculosis Research, Division of Molecular Biology and Human Genetics, Faculty of Medicine and Health Sciences, Stellenbosch University, Cape Town |
| Zaida | Palmer | DSI-NRF Centre of Excellence for Biomedical Tuberculosis Research, South African Medical Research Council Centre for Tuberculosis Research, Division of Molecular Biology and Human Genetics, Faculty of Medicine and Health Sciences, Stellenbosch University, Cape Town |
| Fikiswa | Seti | DSI-NRF Centre of Excellence for Biomedical Tuberculosis Research, South African Medical Research Council Centre for Tuberculosis Research, Division of Molecular Biology and Human Genetics, Faculty of Medicine and Health Sciences, Stellenbosch University, Cape Town |
| Grant | Theron | DSI-NRF Centre of Excellence for Biomedical Tuberculosis Research, South African Medical Research Council Centre for Tuberculosis Research, Division of Molecular Biology and Human Genetics, Faculty of Medicine and Health Sciences, Stellenbosch University, Cape Town |
| Charmaine | Van Der Walt | DSI-NRF Centre of Excellence for Biomedical Tuberculosis Research, South African Medical Research Council Centre for Tuberculosis Research, Division of Molecular Biology and Human Genetics, Faculty of Medicine and Health Sciences, Stellenbosch University, Cape Town |
| Lusanda | Yekani | DSI-NRF Centre of Excellence for Biomedical Tuberculosis Research, South African Medical Research Council Centre for Tuberculosis Research, Division of Molecular Biology and Human Genetics, Faculty of Medicine and Health Sciences, Stellenbosch University, Cape Town |
| **Uganda** | | |
| Alfred | Andama | Makerere University College of Health Sciences, Department of Medicine, Kampala |
| Lucy | Asege | Walimu, Kampala |
| Alice | Bukirwa | Walimu, Kampala |
| David | Katumba | Walimu, Kampala |
| Esther | Kisakye | Walimu, Kampala |
| Wilson | Mangeni | Walimu, Kampala |
| Job | Mukwatamundu | Walimu, Kampala |
| Sandra | Mwebe | Walimu, Kampala |
| Annet | Nakaweesa | Walimu, Kampala |
| Martha | Nakaye | Walimu, Kampala |
| Talemwa | Nalugwa | Walimu, Kampala |
| Irene | Nassuna | Walimu, Kampala |

| First name | Surname | Affiliation |
|---|---|---|
| Irene | Nekesa | Walimu, Kampala |
| Justine | Nyawere | Walimu, Kampala |
| John Baptist | Ssonko | Walimu, Kampala |
| William | Worodria | Walimu, Kampala |
| **Vietnam** | | |
| Hai | Dang | Vietnam National Tuberculosis Program-University of California San Francisco Research Collaboration Unit; Center for Promotion of Advancement of Society, Hanoi |
| Luong | Dinh | Vietnam National Lung Hospital, Hanoi |
| Hang | Do | Hanoi Lung Hospital, Hanoi |
| Tam | Do | Hanoi Lung Hospital, Hanoi |
| Thuong | Do | Vietnam National Lung Hospital, Hanoi |
| Dung | Dao | Hanoi Lung Hospital, Hanoi |
| Ha | Doan | National TB reference Lab/ Vietnam National Lung Hospital, Hanoi |
| Thien | Doan | Hanoi Lung Hospital, Hanoi |
| Huy | Ha | Vietnam National Tuberculosis Program-University of California San Francisco Research Collaboration Unit, Center for Promotion of Advancement of Society, Hanoi |
| Oanh | Lai | Hanoi Lung Hospital, Hanoi |
| Hien | Le | Vietnam National Tuberculosis Program-University of California San Francisco Research Collaboration Unit; Center for Promotion of Advancement of Society, Hanoi |
| Nguyet | Le | National TB reference Lab/ Vietnam National Lung Hospital, Hanoi |
| Anh | Nguyen | Hanoi Lung Hospital, Hanoi |
| Dong | Nguyen | Hanoi Lung Hospital, Hanoi |
| Hanh | Nguyen | Vietnam National Tuberculosis Program-University of California San Francisco Research Collaboration Unit; Center for Promotion of Advancement of Society, Hanoi |
| Hoa | Nguyen | Vietnam National Lung Hospital |
| Hoang | Nguyen | Hanoi Lung Hospital, Hanoi |
| Thanh | Nguyen | Hanoi Lung Hospital, Hanoi |
| Nhung | Nguyen | Vietnam National Tuberculosis Program-University of California San Francisco Research Collaboration Unit, Vietnam National University, Hanoi |
| Yen | Nguyen | Hanoi Lung Hospital, Hanoi |
| Ha | Phan | Vietnam National Tuberculosis Program-University of California San Francisco Research Collaboration Unit, Center for Promotion of Advancement of Society, Hanoi |
| Nam | Pham | Vietnam National Tuberculosis Program-University of California San Francisco Research Collaboration Unit, Hanoi Lung Hospital, Hanoi |
| Thuong | Pham | Hanoi Lung Hospital, Hanoi |

| First name | Surname | Affiliation |
| --- | --- | --- |
| Trang | Trinh | Vietnam National Tuberculosis Program-University of California San Francisco Research Collaboration Unit, Center for Promotion of Advancement of Society, Hanoi |
| Phuong | Vu | Hanoi Lung Hospital, Hanoi |
| Trung | Vu | National TB reference Lab/ Vietnam National Lung Hospital, Hanoi |
| **Zambia** | | |
| Monde | Muyoyeta | Centre for Infectious Disease Research in Zambia (CIDRZ), Lusaka |
| Masuzyo | Chirwa | Centre for Infectious Disease Research in Zambia (CIDRZ), Lusaka |
| Seke | Muzazu | Centre for Infectious Disease Research in Zambia (CIDRZ), Lusaka |
| Brian | Shuma | Centre for Infectious Disease Research in Zambia (CIDRZ), Lusaka |
| Nsala | Sanjase | Centre for Infectious Disease Research in Zambia (CIDRZ), Lusaka |
| Solomon | Chifwambi | Centre for Infectious Disease Research in Zambia (CIDRZ), Lusaka |
| Chileshe | Mukuka | Centre for Infectious Disease Research in Zambia (CIDRZ), Lusaka |
| Pauline | Musumali | Centre for Infectious Disease Research in Zambia (CIDRZ), Lusaka |
| Sibeso | Akashi | Centre for Infectious Disease Research in Zambia (CIDRZ), Lusaka |
| Kachimba | Shamaoma | Centre for Infectious Disease Research in Zambia (CIDRZ), Lusaka |
| Francesca | Silwamba | Centre for Infectious Disease Research in Zambia (CIDRZ), Lusaka |
| Sheba | Nalwamba | Centre for Infectious Disease Research in Zambia (CIDRZ), Lusaka |
| Mathew | Pikiti | Centre for Infectious Disease Research in Zambia (CIDRZ), Lusaka |
| Patrick | Chileshe | Centre for Infectious Disease Research in Zambia (CIDRZ), Lusaka |
| Mike | Phiri | Centre for Infectious Disease Research in Zambia (CIDRZ), Lusaka |
| Abigail | Chunza | Centre for Infectious Disease Research in Zambia (CIDRZ), Lusaka |
| Margret | Chipili | Centre for Infectious Disease Research in Zambia (CIDRZ), Lusaka |
| Andrew | Moono | Centre for Infectious Disease Research in Zambia (CIDRZ), Lusaka |
| Jacinta | Muyaba | Centre for Infectious Disease Research in Zambia (CIDRZ), Lusaka |
| Nyambe | Kakula | Centre for Infectious Disease Research in Zambia (CIDRZ), Lusaka |
| Benson | Jere | Centre for Infectious Disease Research in Zambia (CIDRZ), Lusaka |

| First name | Surname | Affiliation |
|---|---|---|
| Mwiinga | Mwendalubi | Centre for Infectious Disease Research in Zambia (CIDRZ), Lusaka |
| Regina | Banda | Centre for Infectious Disease Research in Zambia (CIDRZ), Lusaka |
| Kella | Siame | Centre for Infectious Disease Research in Zambia (CIDRZ), Lusaka |
| Priscilla | Chisanga | Centre for Infectious Disease Research in Zambia (CIDRZ), Lusaka |
| **USA** | | |
| Robert | Castro | University of California San Francisco, San Francisco, CA |
| Adithya | Cattamanchi | University of California Irvine, Irvine, CA |
| Catherine | Cook | University of California San Francisco, San Francisco, CA |
| Rebecca | Crowder | University of California San Francisco, San Francisco, CA |
| Sophie | Huddart | University of California San Francisco, San Francisco, CA |
| Devan | Jaganath | University of California San Francisco, San Francisco, CA |
| Midori | Kato-Maeda | University of California San Francisco, San Francisco, CA |
| Tessa | Mochizuki | University of California San Francisco, San Francisco, CA |
| Caitlin | Moe | University of California Irvine, Irvine, CA |
| Ruvandhi | Nathavitharana | Beth Israel Deaconess Medical Center, Harvard Medical School, Boston, MA |
| Payam | Nahid | University of California San Francisco, San Francisco, CA |
| Kevin | Nolan | University of California San Francisco, San Francisco, CA |
| Patrick | Phillips | University of California San Francisco, San Francisco, CA |
| Hayley | Poore | University of California Irvine, Irvine, CA |
| Kinari | Shah | University of California San Francisco, San Francisco, CA |
| Brittney | Sweetser | University of California Irvine, Irvine, CA |
| Qiao | Wang | University of California Irvine, Irvine, CA |
| Christina | Yoon | University of California San Francisco, San Francisco, CA |
| **Germany** | | |
| Maria del Mar | Castro Noriega | Heidelberg University Hospital, Heidelberg |
| Margaretha | De Vos | Heidelberg University Hospital, Heidelberg |
| Claudia Maria | Denkinger | Heidelberg University Hospital, Heidelberg |
| Verena | Faehling | Heidelberg University Hospital, Heidelberg |
| Mary | Gaeddert | Heidelberg University Hospital, Heidelberg |
| Ankur | Gupta-Wright | Heidelberg University Hospital, Heidelberg Department of Infectious Diseases, Imperial College London, UK |
| Sonal | Jain | Heidelberg University Hospital, Heidelberg |

| First name | Surname | Affiliation |
|---|---|---|
| Lydia | Holtgrewe | Heidelberg University Hospital, Heidelberg |
| Florian Michael | Marx | Heidelberg University Hospital, Heidelberg |
| Penelope | Papadopoulou | Heidelberg University Hospital, Heidelberg |
| Miriam | Pasinato | Heidelberg University Hospital, Heidelberg |
| Theresa | Pfurtscheller | Heidelberg University Hospital, Heidelberg |
| Pia Anna | Steimer | Heidelberg University Hospital, Heidelberg |
| Stefan Fabian | Weber | Heidelberg University Hospital, Heidelberg |
| Seda | Yerlikaya | Heidelberg University Hospital, Heidelberg |

# Supplementary Methods

## Study Objectives

The primary objectives were to evaluate the diagnostic accuracy of the MiniDock MTB Test (Guangzhou Pluslife Biotech, China) for detecting *Mycobacterium tuberculosis* complex (MTBC) in adolescents and adults with presumed tuberculosis (TB) when using both sputum swab and tongue swab samples, and to assess the test's usability among routine healthcare workers involved in sample collection and TB testing at the participating study sites. Secondary objectives included quantifying the proportion of invalid results, and comparing the diagnostic accuracy of the MiniDock MTB Test and standard comparator tests, including sputum smear microscopy and Xpert MTB/RIF Ultra (Xpert Ultra; Cepheid, Sunnyvale, CA, United States), relative to the microbiological reference standard (MRS).

## Eligibility Criteria

Eligible participants for the accuracy study were aged 12 years or older and met one of the following inclusion criteria: (1) a new or worsening cough lasting at least two weeks, or (2) the presence of at least one TB risk factor combined with a positive result on a WHO-recommended TB screening test. TB risk factors included HIV infection, self-reported close contact with a person with TB, or a history of mining work. WHO-recommended screening tests included either an abnormal chest radiograph or, for people living with HIV, a C-reactive protein (CRP) level greater than 5 mg/L. Exclusion criteria included having received treatment for TB infection or disease within the past 12 months, having taken medications with antimycobacterial activity (e.g., fluoroquinolones) within the previous two weeks, residing more than 20 kilometers from the study site, or expressing unwillingness to return for follow-up assessments or provide informed consent.

Eligible participants for the usability assessment were adults (≥18 years) involved in routine TB care, namely clinicians (e.g., medical doctors, clinical officers), nurses, or laboratory personnel, who were proficient in English or the primary local language, provided informed consent, and had no prior hands-on experience with the MiniDock MTB Test. Exclusion criteria included lay health workers (e.g., community volunteers, adherence supporters) and individuals responsible for operating the MiniDock MTB Test in the accuracy study. Sampling was purposive, designed to reflect the intended end-user group and to capture variation in professional role, clinical experience, and familiarity with TB diagnostic workflows.

## Study Procedures

Biological specimens collected from each study participant included blood, tongue swabs, and sputum.

Blood obtained by finger prick or venipuncture was used for HIV testing and diabetes screening using glycated haemoglobin (HbA1c), as well as two tongue swabs and up to three spot sputum samples.

Tongue swabs were obtained prior to sputum collection by swabbing the dorsum of the tongue continuously for 30 seconds using the swab provided in the MiniDock MTB kit, as per manufacturer's instructions. Whether the first or second tongue swab was used for MiniDock MTB testing was alternated by enrollment day.

Sputum induction with hypertonic saline (3–10%) was performed in participants unable to spontaneously produce sufficient sputum samples. After clearing nasal secretions and receiving an explanation of the procedure, participants inhaled aerosolized saline via nebulizer while seated and breathing normally. Coughing was encouraged to aid sputum production. Nebulization and expectoration were repeated for up to 15 minutes until 3–5 mL of sputum was collected. Participants were monitored for bronchospasm or other adverse effects.

The first sputum sample was used to prepare a sputum swab by rotating the provided swab in the specimen for approximately 15 seconds (10 full rotations), followed by wiping the swab along the inside wall of the sputum container, as per manufacturer instructions. The remainder of the first sputum sample was used for Ultra testing. The second and third sputum samples were processed using standard decontamination with N-acetyl-L-cysteine/sodium hydroxide for subsequent microbiological analysis.

Each usability session was facilitated by a trained moderator and observer and involved participants independently performing the MiniDock MTB Test using only the provided instructions for use (IFU), with no additional training. Procedures included task observation using a structured checklist, a think-aloud protocol (when consent was provided), interpretation of simulated test results, and a post-test survey incorporating the System Usability Scale (SUS), along with open-ended questions on acceptability and user feedback. Observers recorded task completion, errors, and operational challenges using a checklist derived from the IFU and refined through pilot testing and consultation with the test developer. Regular debriefing sessions with site teams were held to promote procedural consistency and ensure data quality.

## Index Test

Index testing used design-locked, good manufacturer practices (GMP)-manufactured versions of the MTB Nucleic Acid Test Card (MiniDock MTB Test), Pluslife Thermolyse, and the Pluslife Integrated Nucleic Acid Testing Device (MiniDock Ultra). Catalog numbers, lot numbers, and serial numbers are listed in Table S2.

All assays were conducted according to the manufacturer's IFU (see Supplementary File 1). Operators were trained via a standardized online program.

Device-level quality control procedures included running one positive and one negative control, supplied by the manufacturer, on each MiniDock unit at the start of the study and after every 100 participants enrolled.

## Comparator Tests

Light emitting diode (LED) fluorescence microscopy was performed on two replicate smears prepared from digested and decontaminated sputum. The majority of smears were stained with auramine; however, approximately 7% were stained using the Ziehl–Neelsen (ZN) method, in accordance with WHO-recommended protocols.[1] A smear was considered positive when at least one acid-fast bacillus (AFB) was observed in 100 fields.

Xpert Ultra was performed according to the instructions of the manufacturer. Results were recorded as positive for MTB (semi-quantitative grade of 'very low' or higher), negative for MTB, or invalid/error.

## Reference Standard

The MRS for TB diagnosis was based on culture results using liquid mycobacteria growth indicator tube (MGIT) media (Becton Dickinson, Franklin Lakes, NJ, USA; SKU: 445870, GTIN: 00382904458706).[2] Two replicate cultures were performed for each participant. A participant was classified as TB-positive if at least one culture was positive for MTBC, and as TB-negative if both cultures were negative. Participants with contaminated results in one or both cultures and no positive results were classified as having indeterminate TB and were excluded from diagnostic accuracy analyses.

As a secondary reference standard, a composite reference standard (CRS) was used. The CRS classified TB status for participants with an indeterminate MRS result based on additional TB test results obtained through routine care, as well as findings from repeat symptom assessment, chest X-ray (CXR), and sputum testing conducted 2–4 months after enrollment. Based on these data, two reviewers independently classified participants as TB likely, TB unlikely, or TB indeterminate. Any discrepancies between reviewers were resolved by a third reviewer.

## Outcome Measures

The primary outcomes were the diagnostic accuracy of the initial MiniDock MTB test, as measured by its sensitivity and specificity for detecting MTBC, compared to the MRS, and the usability of the assay, as assessed using the SUS.

Secondary outcomes included the proportion of invalid MiniDock MTB results and the sensitivity and specificity differences relative to the MRS between MiniDock MTB and the two-routine sputum-based comparator tests: smear microscopy and Xpert Ultra.

## Sample Size Estimation

The study aimed to evaluate the accuracy of the index test in a cohort of 1,000 participants with presumptive pulmonary TB. Assuming a TB prevalence of approximately 20%, this minimum sample size was selected to ensure reasonable precision in estimating diagnostic accuracy, particularly sensitivity and specificity, with 95% confidence intervals (CIs). Based on expected test performance, the width of the 95% CI for sensitivity estimates ranging from 80% to 90% was expected to vary between 8.8% and 11.5%, while the CI width for specificity estimates between 95% and 98% was expected to range from 2.1% to 3.2%.

No sample size calculations were made for the usability assessment. We sought to include a minimum of 15 participants, with a target of 8-10 participants per country (maximum of 20 in total), in accordance with the WHO Technical Specifications Series (TSS)-17.[3]

## Data Analysis

Sensitivity and specificity were calculated against the MRS, with their exact binomial 95% confidence intervals (CIs), both overall and within key subgroups. Differences between the index and comparator tests were assessed using McNemar's test for paired proportions.

Indeterminate results in the reference standard and inconclusive test results were rare; these data were therefore excluded from the complete case analysis and assumed to be missing completely at random (MCAR).[4]

Pre-specified sensitivity analyses included repeat index test results and consideration of “Trace” sputum Xpert Ultra results as positive. Concordance between MiniDock MTB and Xpert Ultra was assessed both overall and by Xpert Ultra semi-quantitative grade. Secondary analyses were conducted using the CRS.

Confidence intervals were not adjusted for multiplicity and should not be interpreted as formal hypothesis tests. No multiplicity adjustment was applied for the sensitivity analyses, as each comparison addressed a distinct, pre-specified objective. All statistical analyses were performed using R version 4.3.1 (R Foundation for Statistical Computing, Vienna, Austria).

Participant characteristics and survey responses were summarized through descriptive statistics using STATA version 14 (StataCorp, 2015). Coding of the qualitative data was conducted iteratively to identify recurring patterns, with special attention to usability barriers, error-prone steps, preliminary acceptability and user recommendations for improvement. Themes were organized to align with usability domains and acceptability.

We also conducted a classification of errors and usability issues by severity, considering the potential for harm (actions that result in invalid, inaccurate results, and/or safety risk to the user).

- Critical: Errors that cause safety issues for the user and/or could result in invalid or inaccurate results.
- Major: Issues that slow task completion, introduce human error during the task, and/or present an elevated safety risk to the user. Users must recover from the error, which further delays their progress.
- Minor: Issues that cause annoyances to the user but do not prevent task completion or increase safety risk.

## STARD Reporting Checklist

| | Item Description | Location (or reason for not reporting) |
|---|---|---|
| **Title or abstract** | | |
| 1. Identification as a study of diagnostic accuracy | Identification as a study of diagnostic accuracy using at least one measure of accuracy (such as sensitivity, specificity, predictive values, or AUC). | Abstract |
| **Abstract** | | |
| 2. Abstract | Structured summary of study design, methods, results and conclusions (for specific guidance, see STARD for Abstracts). | Abstract |
| **Introduction** | | |
| 3. Background | Scientific and clinical background, including the intended use and clinical role of the index test. | Introduction, para. 1-3 |
| 4. Objectives | Study objectives and hypotheses. | Introduction, para. 4<br>Supplementary Methods, § Study Objectives |
| **Methods** | | |
| 5. Study design | Whether data collection was planned before the index test and reference standard were | Methods, § Study Design and Setting |

| | | |
|---|---|---|
| | performed (prospective study) or after (retrospective study). | |
| Participants | | |
| 6. Eligibility criteria | Eligibility criteria. | Methods, § Study Participants<br>Supplementary Methods, § Eligibility Criteria |
| 7. Identifying eligible participants | On what basis potentially eligible participants were identified (such as symptoms, results from previous tests, inclusion in registry). | Methods, § Study Participants |
| 8. Setting, location, and dates | Where and when potentially eligible participants were identified (setting, location and dates). | Methods, § Study Participants<br>Table S1 |
| 9. Consecutive, random or convenience series | Whether participants formed a consecutive, random or convenience series. | Methods, § Study Participants |
| Test Methods | | |
| 10a. Index test | Index test, in sufficient detail to allow replication. | Methods, § Study Procedures<br>Methods, § Index test<br>Supplementary Methods, § Study Procedures<br>Supplementary Methods, § Index test<br>Supplementary File |
| 10b. Reference standard | Reference standard, in sufficient detail to allow replication. | Methods, § Study Procedures<br>Methods, § Reference Standard<br>Supplementary Methods, § Study Procedures<br>Supplementary Methods, § Reference Standard |
| 11. Reference standard rationale | Rationale for choosing the reference standard (if alternatives exist). | Methods, § Reference Standard |
| 12a. Index test cut-offs or categories | Definition of and rationale for test positivity cut-offs or result categories of the index test, distinguishing prespecified from exploratory. | Not applicable: developer-defined qualitative result categories |
| 12b. Reference standard cut-offs or categories | Definition of and rationale for test positivity cut-offs or result categories of the reference standard, distinguishing prespecified from exploratory. | Not applicable: MGIT result categories (positive/negative) defined according to WHO-recommended procedures |

| | | |
|---|---|---|
| 13a. Information available to performers or readers of the index test | Whether clinical information and reference standard results were available to the performers or readers of the index test. | Methods, § Index test |
| 13b. Information available to reference standard assessors | Whether clinical information and index test results were available to the assessors of the reference standard. | Methods, § Reference Standard |
| Analysis | | |
| 14. Analysis methods | Methods for estimating or comparing measures of diagnostic accuracy. | Methods, § Statistical Analysis |
| 15. Indeterminate results | How indeterminate index test or reference standard results were handled. | Methods, § Statistical Analysis<br>Supplementary Methods, § Reference Standard |
| 16. Missing data | How missing data on the index test and reference standard were handled. | Methods, § Statistical Analysis<br>Supplementary Methods, § Reference Standard |
| 17. Variability | Any analyses of variability in diagnostic accuracy, distinguishing prespecified from exploratory. | Methods, § Statistical Analysis |
| 18. Intended sample size | Intended sample size and how it was determined. | Methods, § Statistical Analysis<br>Supplementary Methods, § Sample Size Estimation |
| **Results** | | |
| Participants | | |
| 19. Participant flow diagram | Flow of participants, using a diagram. | Figure 1 |
| 20. Baseline characteristics | Baseline demographic and clinical characteristics of participants. | Table 1, Table S3 |
| 21a. Participants with the target condition | Distribution of severity of disease in those with the target condition. | Table 1, Table S3 |
| 21b. Participants without the target condition | Distribution of alternative diagnoses in those without the target condition. | Not reported: alternative diagnoses were not systematically collected |
| 22. Time interval | Time interval and any clinical interventions between index test and reference standard. | Methods, § Study Procedures |
| Test Results | | |
| 23. Index test and reference standard results | Cross tabulation of the index test results (or their distribution) by the results of the reference standard. | Figures 2-3 |

| | | |
|---|---|---|
| 24. Estimates of accuracy | Estimates of diagnostic accuracy and their precision (such as 95% CIs). | Figures 2-3 |
| 25. Adverse events | Any adverse events from performing the index test or the reference standard. | Results, para. 12 |
| **Discussion** | | |
| 26. Limitations | Study limitations, including sources of potential bias, statistical uncertainty and generalizability. | Discussion, para. 6 |
| 27. Implications for Practice | Implications for practice, including the intended use and clinical role of the index test. | Discussion, para. 4 |
| **Other information** | | |
| 28. Registration | Registration number and name of registry. | Methods, §Ethics Statement |
| 29. Protocol | Where the full study protocol can be accessed. | Methods, §Ethics Statement |
| 30. Funding | Sources of funding and other support; role of funders. | Sources of support |

## Supplementary Figures

**Figure S1:** Distribution of responses to individual system usability scale (SUS) items regarding the use of MiniDock MTB among health care workers in India (n=10) and South Africa (n=8).

Bar plot showing the count of user responses (n = 18) for each of the 10 SUS items, grouped by Likert scale option (1 = Strongly Disagree to 5 = Strongly Agree).

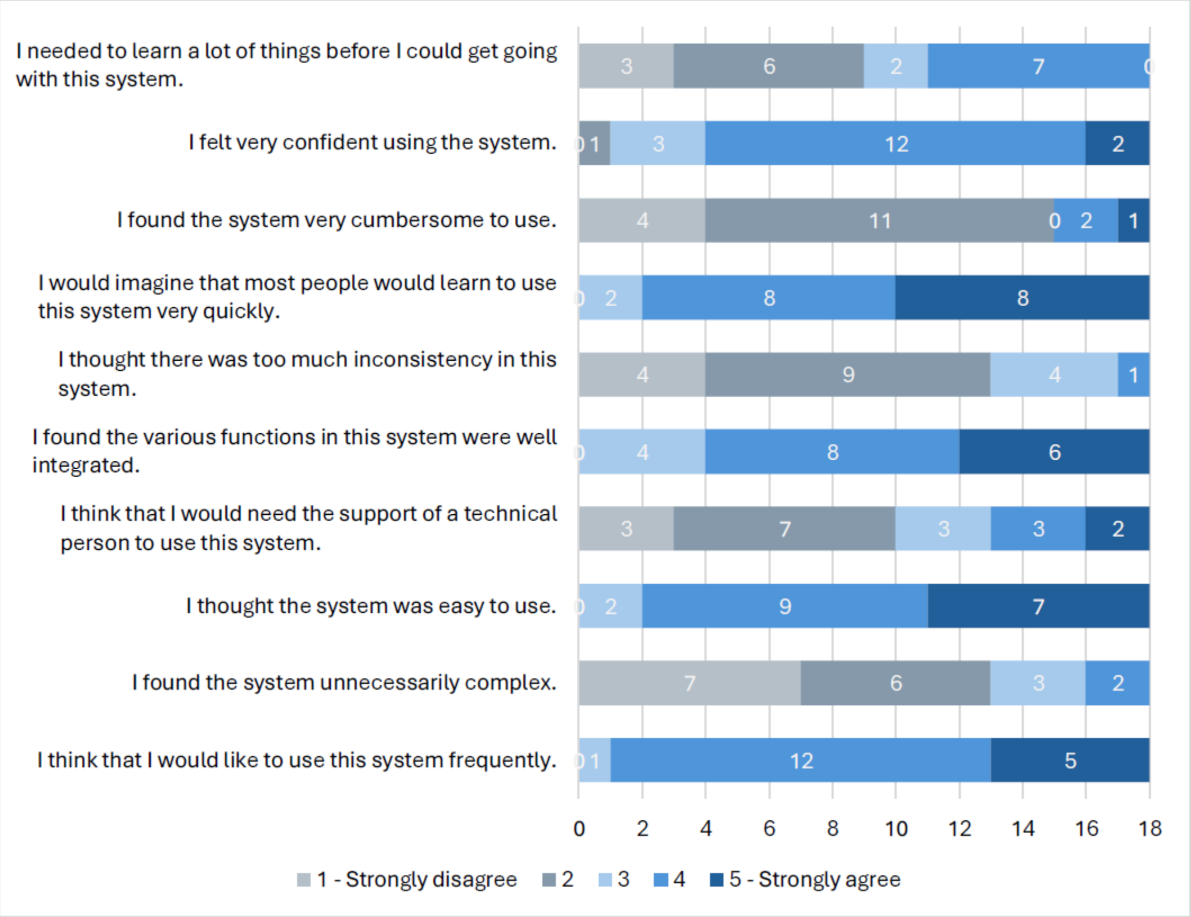

**Figure S2:** Acceptability of MiniDock MTB

Bar plot showing the count of user responses (n = 18) for acceptability, grouped by Likert scale option (1 = Strongly Disagree to 5 = Strongly Agree).

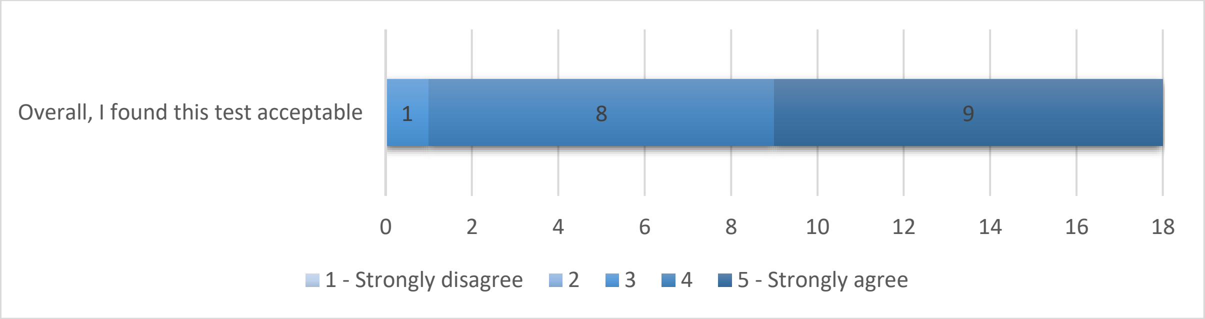

## Supplementary Tables

**Table S1:** Enrollment sites and ethics committees*

| Country | Enrollment site(s) | Ethics committee and approval number | Enrolment dates |
|---|---|---|---|
| India | 1. Christian Medical College (CMC) Pulmonary Outpatient Department, Vellore, Tamil Nadu<br>2.Primary care clinic in Vellore (CHAD), Vellore, Tamil Nadu<br>3. Chittoor (CMC satellite campus), Chittoor, Andhra Pradesh | Christian Medical College Institutional Review Board (13256) | SEP 30, 2024 to JAN 24, 2025 |
| Nigeria | 1. General Hospital Nyanya, Abuja<br>2.Township Primary Healty Clinic, Gwagwalada, Abuja | Federal Capital Territory Health Research Ethics Committee (FHREC/2023/01/125/20-07-23) | JAN 13, 2025 to MAR 27, 2025 |
| Philippines | 1. City Health Office 1, Dasmariñas<br>2. De La Salle University Medical Center, Dasmariñas<br>3. Community Health Department, Silang Bulihan Municipality, Silang, Cavite<br>4. Community Health Department, General Mariano Alvarez (GMA) Municipality, Cavite | De La Salle Health Sciences Institute Independent Ethics Committee (2020-33-02-A) | SEP 30, 2024 to MAR 31, 2025 |
| South Africa | 1. Scottsdene Primary Care Clinic Kfaaifontain, Capetown<br>2. Wallacedene Primary Care Clinic Wallace Dene, Capetown<br>3. Kfaaifontain Community Health Center Kfaaifontain, Capetown | Stellenbosch University Health Research Ethics Committee (M20/07/020) | SEP 30, 2024 to JAN 31, 2025 |
| Uganda | 1. Mulago Outpatient Department, Kampala<br>2. Kisenyi Health Center, Kampala | Makerere University, College of Health Sciences, School of Medicine, Research Ethics Committee (2020-182); Uganda National Council for Science and Technology (HS1482ES) | SEP 30, 2024 to MAR 31, 2025 |
| Vietnam | 1. Outpatient departments, Hanoi Lung Hospital, Hanoi | Ministry of Health Ethical Committee for National Biological Medical Research (94/CN-HĐĐĐ); National Lung Hospital Ethical Committee for Biological Medical Research (566/2020/NCKH); Hanoi Lung Hospital Science and Technology Initiative Committee (22/BVPHN) | SEP 12, 2024 to MAR 31, 2025 |
| Zambia | 1. Chawama Hospital, Lusaka<br>2. Kanyama Hospital, Lusaka | University of Zambia Biomedical Research Ethics Committee (4197-2023); National Health Research Authority (00008/06/09/2023) | OCT 23, 2024 to MAR 31, 2025 |

*This study was also approved by the University of California, San Francisco Institutional Review Board (20-32670), and the University of Heidelberg Ethics Committee of the Medical Faculty (S-539/2020).

**Table S2:** Kit lot numbers and instrument serial numbers of the Guangzhou Pluslife Biotech equipment and reagents per country.

| Item(s) | India | Nigeria | Philippines | South Africa | Uganda | Vietnam | Zambia |
|---|---|---|---|---|---|---|---|
| MTB Nucleic Acid Test Card (MiniDock® MTB Test, catalog number: RM1012202-10) Lot Number | 20240704<br>20240703 | 20240704<br>20240703 | 20240704<br>20240703 | 20240704<br>20240703 | 20240704<br>20240703 | 20240704<br>20240703 | 20240704<br>20240703 |
| Mycobacterium Tuberculosis Nucleic Acid Assay Control (Catalog number: CRM1010602-1) (1 set/box) Lot Number | 20240801 | | | | | | |
| Integrated Nucleic Acid Testing Device (MiniDock® Ultra, model: PM001 Ultra) Serial Number | 2429070024<br>2429070025<br>2429070026<br>2429070027 | 2429070048<br>2429070049<br>2429070050<br>2429070051 | 2429070052<br>2429070053<br>2429070054<br>2429070055<br>2429070056<br>2429070057<br>2429070058<br>2429070059<br>2429070148 | 2429070040<br>2429070041<br>2429070042<br>2429070043<br>2429070044<br>2429070045<br>2429070046<br>2429070047 | 2429070016<br>2429070017<br>2429070018<br>2429070019<br>2429070020<br>2429070021<br>2429070022<br>2429070023 | 2429070036<br>2429070037<br>2429070038<br>2429070039 | 2429070028<br>2429070029<br>2429070030<br>2429070031<br>2429070032<br>2429070033<br>2429070034<br>2429070035 |
| Pluslife Thermolyse (Model: WD002) Serial Number | WD2Q070037<br>WD2Q070028 | WD2Q070052<br>WD2Q070061 | WD2Q070070<br>WD2Q070035 | WD20070044<br>WD20070067<br>WD20070063<br>WD20070026 | WD2Q070051<br>WD2Q070045 | WD2Q070060<br>WD2Q70075 | WD2Q070029<br>WD2Q070040 |
| 5 Ports HUB (Model: HB001) Serial Number | 022239010038 | 022239010061 | 022239010040 | 022239010026<br>022239010027 | 022239010028<br>022237010106 | 022239010060 | 022239010029 |
| Pluslife software version | SV 4.3.1 | | | | | | |

**Table S3:** Demographic and clinical characteristics of study participants*

| | All | IN | NG | PH | SA | UG | VN | ZM |
|---|---|---|---|---|---|---|---|---|
| **Total Enrolled,** *N (%)* | 1380 (100%) | 210 (15.2%) | 96 (7.0%) | 182 (13.2%) | 270 (19.6%) | 262 (19.0%) | 180 (13.0%) | 180 (13.0%) |
| **Female,** *N (%)* | 603 (43.7%) | 91 (43.3%) | 41 (42.7%) | 91 (50.0%) | 137 (50.7%) | 102 (38.9%) | 81 (45.0%) | 60 (33.3%) |
| **Age,** *median (IQR)* | 41 (29–54) | 51 (36–61) | 35 (26–45) | 50 (33–61) | 37 (28–46) | 34 (23–45) | 53 (40–64) | 37 (29–46) |
| **Age Category,** *N (%)* | | | | | | | | |
| 12-17 years | 49 (3.6%) | 8 (3.8%) | 2 (2.1%) | 5 (2.7%) | 10 (3.7%) | 21 (8.0%) | 2 (1.1%) | 1 (0.6%) |
| 18-34 years | 461 (33.4%) | 35 (16.7%) | 44 (45.8%) | 48 (26.4%) | 106 (39.3%) | 113 (43.1%) | 34 (18.9%) | 81 (45.0%) |
| 35-64 years | 735 (53.3%) | 127 (60.5%) | 47 (49.0%) | 101 (55.5%) | 147 (54.4%) | 122 (46.6%) | 100 (55.6%) | 91 (50.6%) |
| ⩾65 years | 135 (9.8%) | 40 (19.0%) | 3 (3.1%) | 28 (15.4%) | 7 (2.6%) | 6 (2.3%) | 44 (24.4%) | 7 (3.9%) |
| **Symptomatic** [a]**,** *N (%)* | 1342 (97.2%) | 207 (98.6%) | 96 (100%) | 179 (98.4%) | 258 (95.6%) | 243 (92.7%) | 180 (100%) | 179 (99.4%) |
| **Cough ≥ 2 weeks,** *N (%)* | 1313 (95.1%) | 207 (98.6%) | 96 (100%) | 175 (96.2%) | 247 (91.5%) | 236 (90.1%) | 180 (100%) | 172 (95.6%) |
| **Previous history of TB,** *N (%)* | | | | | | | | |
| Yes | 236 (17.1%) | 19 (9.0%) | 6 (6.3%) | 42 (23.1%) | 69 (25.6%) | 26 (9.9%) | 38 (21.1%) | 36 (20.0%) |
| No | 1141 (82.7%) | 191 (91.0%) | 89 (92.7%) | 140 (76.9%) | 200 (74.1%) | 236 (90.1%) | 141 (78.3%) | 144 (80.0%) |
| Unknown | 3 (0.2%) | 0 (0%) | 1 (1.0%) | 0 (0%) | 1 (0.4%) | 0 (0%) | 1 (0.6%) | 0 (0%) |
| **HIV status** [b]**,** *N (%)* | | | | | | | | |
| Positive | 255 (18.5%) | 1 (0.5%) | 6 (6.3%) | 2 (1.1%) | 96 (35.6%) | 76 (29.0%) | 5 (2.8%) | 69 (38.3%) |
| Negative | 1119 (81.1%) | 204 (97.1%) | 90 (93.8%) | 180 (98.9%) | 174 (64.4%) | 186 (71.0%) | 174 (96.7%) | 111 (61.7%) |
| Unknown | 6 (0.4%) | 5 (2.4%) | 0 (0%) | 0 (0%) | 0 (0%) | 0 (0%) | 1 (0.6%) | 0 (0%) |
| **Receiving ART among HIV-positive,** *N (%)* | | | | | | | | |
| Yes | 188 (73.7%) | 1 (100%) | 5 (83.3%) | 0 (0%) | 66 (68.8%) | 62 (81.6%) | 4 (80.0%) | 50 (72.5%) |
| No | 49 (19.2%) | 0 (0%) | 1 (16.7%) | 0 (0%) | 23 (24.0%) | 6 (7.9%) | 0 (0%) | 19 (27.5%) |
| Unknown | 18 (7.1%) | 0 (0%) | 0 (0%) | 2 (100.0%) | 7 (7.3%) | 8 (10.5%) | 1 (20.0%) | 0 (0%) |
| **Diabetes status** [c]**,** *N (%)* | | | | | | | | |
| Positive | 187 (13.6%) | 57 (27.1%) | 6 (6.3%) | 23 (12.6%) | 27 (10.0%) | 33 (12.6%) | 18 (10.0%) | 23 (12.8%) |
| Negative | 1193 | 153 | 90 | 159 | 243 | 229 | 162 | 157 |

| | | | | | | | | |
|---|---|---|---|---|---|---|---|---|
| | (86.5%) | (72.9%) | (93.8%) | (87.4%) | (90.0%) | (87.4%) | (90.0%) | (87.2%) |
| **Ability-expectorate sputum,** *N (%)* | | | | | | | | |
| Expectorated | 1236 (89.6%) | 189 (90%) | 96 (100%) | 122 (67%) | 252 (93.3%) | 217 (82.8%) | 180 (100%) | 180 (100%) |
| Induced | 144 (10.4%) | 21 (10.0%) | 0 (0%) | 60 (33.0%) | 18 (6.7%) | 45 (17.2%) | 0 (0%) | 0 (0%) |
| **Sputum smear status,** *N (%)* | | | | | | | | |
| Positive | 144 (10.4%) | 6 (2.9%) | 29 (30.2%) | 19 (10.4%) | 18 (6.7%) | 47 (17.9%) | 7 (3.9%) | 18 (10.0%) |
| Negative | 1236 (89.6%) | 204 (97.1%) | 67 (69.8%) | 163 (89.6%) | 252 (93.3%) | 215 (82.1%) | 173 (96.1%) | 162 (90.0%) |
| **Sputum Ultra status** [d]**,** *N (%)* | | | | | | | | |
| Positive | 213 (15.7%) | 11 (5.2%) | 30 (31.6%) | 28 (15.4%) | 41 (15.6%) | 55 (21.2%) | 18 (10.6%) | 30 (16.8%) |
| High | 88 (41.3%) | 2 (18.2%) | 18 (60.0%) | 8 (28.6%) | 21 (51.2%) | 25 (45.5%) | 4 (22.2%) | 10 (33.3%) |
| Medium | 53 (24.9%) | 4 (36.4%) | 7 (23.3%) | 6 (21.4%) | 10 (24.4%) | 16 (29.1%) | 3 (16.7%) | 7 (23.3%) |
| Low | 49 (23.0%) | 4 (36.4%) | 3 (10.0%) | 8 (28.6%) | 7 (17.1%) | 10 (18.2%) | 8 (44.4%) | 9 (30.0%) |
| Very Low | 23 (10.8%) | 1 (9.1%) | 2 (6.7%) | 6 (21.4%) | 3 (7.3%) | 4 (7.3%) | 3 (16.7%) | 4 (13.3%) |
| Negative | 1144 (82.9%) | 199 (94.8%) | 65 (67.7%) | 153 (84.1%) | 221 (81.9%) | 205 (78.2%) | 152 (84.4%) | 149 (82.8%) |
| Trace | 16 (1.2%) | 0 (0.0%) | 1 (1.0%) | 0 (0.0%) | 5 (1.9%) | 2 (0.8%) | 7 (3.9%) | 1 (0.6%) |
| Unknown | 7 (0.5%) | 0 (0.0%) | 0 (0.0%) | 1 (0.6%) | 3 (1.1%) | 0 (0.0%) | 3 (1.7%) | 0 (0.0%) |
| **MRS status,** *N (%)* | | | | | | | | |
| Positive | 226 (16.4%) | 10 (4.8%) | 27 (28.1%) | 28 (15.4%) | 51 (18.9%) | 59 (22.5%) | 19 (10.6%) | 32 (17.8%) |
| Negative | 1074 (77.8%) | 187 (89.1%) | 65 (67.7%) | 141 (77.5%) | 205 (75.9%) | 181 (69.1%) | 150 (83.3%) | 145 (80.6%) |
| Unknown | 80 (5.8%) | 13 (6.2%) | 4 (4.2%) | 13 (7.1%) | 14 (5.2%) | 22 (8.4%) | 11 (6.1%) | 3 (1.7%) |

Abbreviations: IN: India; NG: Nigeria; PH: Philippines; SA: South Africa; UG: Uganda; VN: Viet Nam; ZA: Zambia; IQR: inter-quartile range; TB: tuberculosis; ART: antiretroviral therapy; MRS: microbiological reference standard

[*] All variables shown without an "Unknown" row were complete (i.e., no missing or indeterminate values).

[a] Symptomatic is defined as any one of the following symptoms: cough ≥ 2 weeks, fever, weight loss, night sweats

[b] HIV positivity defined by self-reported positivity or positive HIV test at baseline.

[c] Diabetes defined by self-reported history of diabetes, currently taking diabetes medications or insulin, or an hba1c result >=6.5%.

[d] Ultra considered positive with semi-quantification result of very low or higher; unknown refers to invalid/error results

**Table S4:** MiniDock MTB invalid rates, overall and by country*

| | **All**<br>n/N (%) | **IN**<br>n/N (%) | **NG**<br>n/N (%) | **PH**<br>n/N (%) | **SA**<br>n/N (%) | **UG**<br>n/N (%) | **VN**<br>n/N (%) | **ZA**<br>n/N (%) |
|---|---|---|---|---|---|---|---|---|
| Sputum initial test | 15/1380 (1.1%) | 1/210 (0.5%) | 1/96 (1%) | 0/182 (0%) | 2/270 (0.7%) | 7/262 (2.7%) | 4/180 (2.2%) | 0/180 (0%) |
| Sputum repeat test | 5/15 (33.3%) | 1/1 (100%) | 1/1 (100%) | - | 0/2 (0%) | 2/7 (28.6%) | 1/4 (25%) | - |
| Tongue swab initial test | 10/1380 (0.7%) | 0/210 (0%) | 0/96 (0%) | 0/182 (0%) | 3/270 (1.1%) | 3/262 (1.1%) | 3/180 (1.7%) | 1/180 (0.6%) |
| Tongue swab repeat test | 0/10 (0%) | - | - | - | 0/3 (0%) | 0/3 (0%) | 0/3 (0%) | 0/1 (0%) |

* Invalid results refer to tests that did not yield a valid diagnostic outcome.

Abbreviations: IN: India, NG: Nigeria, PH: Philippines, SA: South Africa, UG: Uganda, VN: Vietnam, ZA: Zambia.

**Table S5:** Diagnostic accuracy compared to MRS, including repeat test results

| | **Sensitivity**<br>n/N, % (95% CI) | **Specificity**<br>n/N, % (95% CI) |
|---|---|---|
| **Sputum MiniDock MTB** | 193/225, 85.8% (80.5–90.1) | 1044/1070, 97.6% (96.5–98.4) |
| **Tongue swab MiniDock MTB** | 180/226, 79.6% (73.8–84.7) | 1069/1074, 99.5% (98.9–99.8) |

Abbreviations: MRS: microbiological reference standard, CI: confidence interval.

**Table S6:** Diagnostic accuracy compared to CRS*

| | **Sensitivity** n/N, % (95% CI) | **Specificity** n/N, % (95% CI) |
|---|---|---|
| **Sputum MiniDock MTB** | 209/273, 76.6% (71.1–81.5) | 847/861, 98.4% (97.3–99.1) |
| **Tongue swab MiniDock MTB** | 186/275, 67.6% (61.8–73.1) | 863/864, 99.9% (99.4–100.0) |

* Exclusions among participants with valid CRS results (N = 1,145): invalid sputum MiniDock MTB (n = 1) and invalid tongue swab MiniDock MTB (n = 6).
Abbreviations: CRS: composite reference standard, CI: confidence interval.

**Table S7:** Head-to-head diagnostic accuracy comparison between MiniDock MTB and conventional sputum-based TB tests against CRS*

| | Sensitivity<br>n/N, % (95% CI) | Specificity<br>n/N, % (95% CI) |
|---|---|---|
| **Sputum swab MiniDock MTB** | 200/258, 77.5% (71.9–82.5) | 841/855, 98.4% (97.3–99.1) |
| **Sputum Xpert Ultra**** | 211/258, 81.8% (76.5–86.3) | 855/855, 100.0% (99.6–100.0) |
| **Difference (%, 95% CI)** | -4.3% (-7.7 to -0.8) | -1.6% (-2.6 to -0.7) |
| **Sputum swab MiniDock MTB** | 200/258, 77.5% (71.9–82.5) | 841/855, 98.4% (97.3–99.1) |
| **Sputum Microscopy** | 139/258, 53.9% (47.6–60.1) | 853/855, 99.8% (99.2–100.0) |
| **Difference (%, 95% CI)** | 23.6% (17.9 to 29.4) | -1.4% (-2.4 to -0.4) |
| **Tongue swab MiniDock MTB** | 180/258, 69.8% (63.8–75.3) | 854/855, 99.9% (99.4–100.0) |
| **Sputum Xpert Ultra** | 211/258, 81.8% (76.5–86.3) | 855/855, 100.0% (99.6–100.0) |
| **Difference (%, 95% CI)** | -12.0% (-16.8 to -7.2) | -0.1% (-0.5 to 0.2) |
| **Tongue swab MiniDock MTB** | 180/258, 69.8% (63.8–75.3) | 854/855, 99.9% (99.4–100.0) |
| **Sputum Microscopy** | 139/258, 53.9% (47.6–60.1) | 853/855, 99.8% (99.2–100.0) |
| **Difference (%, 95% CI)** | 15.9% (10.4 to 21.3) | -0.1% (-0.4 to 0.6) |

* Analysis includes only participants with valid results on all four tests.
** Analysis treats sputum Ultra “trace” results as negatives.
Abbreviations: CRS: composite reference standard, CI: confidence interval.

**Table S8:** Sensitivity analysis of the head-to-head diagnostic accuracy comparison between MiniDock MTB and sputum Xpert Ultra against MRS, including Ultra trace results

| | **Sensitivity** n/N, % (95% CI) | **Specificity** n/N, % (95% CI) |
|---|---|---|
| **Sensitivity analysis #1: Initial sputum Xpert Ultra "trace" result classified as TB-negative*** | | |
| **Sputum MiniDock MTB** | 191/223, 85.7% (80.4-90.0) | 1031/1056, 97.6% (96.5-98.5) |
| **Sputum Xpert Ultra** | 195/223, 87.4% (82.4-91.5) | 1046/1056, 99.1% (98.3-99.5) |
| **Difference (%, 95% CI)** | -1.8% (-5.3 to +1.7) | -1.4% (-2.5 to -0.4) |
| **Tongue swab MiniDock MTB** | 177/223, 79.4% (73.5-84.5) | 1051/1056, 99.5% (98.9-99.8) |
| **Sputum Xpert Ultra** | 195/223, 87.4% (82.4-91.5) | 1046/1056, 99.1% (98.3-99.5) |
| **Difference (%, 95% CI)** | -8.1% (-12.9 to -3.3) | +0.5% (-0.3 to +1.2) |
| **Sensitivity analysis #2: Initial sputum Xpert Ultra "trace" result classified as TB-positive*** | | |
| **Sputum MiniDock MTB** | 191/223, 85.7% (80.4-90.0) | 1031/1056, 97.6% (96.5-98.5) |
| **Sputum Xpert Ultra** | 200/223, 89.7% (84.9-93.3) | 1038/1056, 98.3% (97.3-99.0) |
| **Difference (%, 95% CI)** | -4.0% (-7.6 to -0.5) | -0.7% (-1.8 to +0.4) |
| **Tongue swab MiniDock MTB** | 177/223, 79.4% (73.5-84.5) | 1051/1056, 99.5% (98.9-99.8) |
| **Sputum Xpert Ultra** | 200/223, 89.7% (84.9-93.3) | 1038/1056, 98.3% (97.3-99.0) |
| **Difference (%, 95% CI)** | -10.3% (-15.3 to -5.3) | +1.2% (+0.3 to +2.1) |
| **Sensitivity analysis #3: Initial sputum Xpert Ultra trace result repeated**** | | |
| **Sputum MiniDock MTB** | 191/223, 85.7% (80.4-90.0) | 1031/1055, 97.7% (96.6-98.5) |
| **Sputum Xpert Ultra** | 199/223, 89.2% (84.4-93.0) | 1041/1055, 98.7% (97.8-99.3) |
| **Difference (%, 95% CI)** | -3.6% (-7.3 to +0.1) | -0.9% (-2.0 to +0.09) |
| **Tongue swab MiniDock MTB** | 177/223, 79.4% (73.5-84.5) | 1050/1055, 99.5% (98.9-99.8) |
| **Sputum Xpert Ultra** | 199/223, 89.2% (84.4-93.0) | 1041/1055, 98.7% (97.8-99.3) |
| **Difference (%, 95% CI)** | -9.9% (-14.8 to -5.0) | +0.9% (+0.04 to +1.7) |

* Includes 13 participants whose initial sputum Xpert Ultra results were "trace."
** Includes 12 participants with initial sputum Xpert Ultra "trace" results who had a valid repeat sputum Xpert Ultra result.
Abbreviations: MRS: microbiological reference standard, CI: confidence interval.

**Table S9:** Concordance between sputum MiniDock MTB and sputum Xpert Ultra test results, overall and by Xpert Ultra semi-quantitative grade.

| **Characteristic** | **Positive concordance** n/N, % (95% CI) | **Negative concordance** n/N, % (95% CI) |
|---|---|---|
| **Overall** (excluding Trace) | 197/211, 93.4% (89.1–96.3) | 1111/1131, 98.2% (97.3–98.9) |
| **Overall** (including Trace) | 203/227, 89.4% (84.7–93.1) | 1111/1131, 98.2% (97.3–98.9) |
| **Xpert Ultra semi-quantitative grade** | | |
| High | 87/87, 100.0% (95.8–100.0) | - |
| Medium | 51/52, 98.1% (89.7–100.0) | - |
| Low | 43/49, 87.8% (75.2–95.4) | - |
| Very low | 16/23, 69.6% (47.1–86.8) | - |
| Trace | 6/16, 37.5% (15.2–64.6) | - |

Abbreviations: CI: confidence interval.

**Table S10:** Concordance between tongue swab MiniDock MTB and sputum Xpert Ultra test results, overall and by Xpert Ultra semi-quantitative grade.

| **Characteristic** | **Positive concordance** n/N, % (95% CI) | **Negative concordance** n/N, % (95% CI) |
|---|---|---|
| **Overall** (excluding Trace) | 179/213, 84.0% (78.4–88.7) | 1128/1134, 99.5% (98.9–99.8) |
| **Overall** (including Trace) | 181/229, 79.0% (73.2–84.1) | 1128/1134, 99.5% (98.9–99.8) |
| **Xpert Ultra semi-quantitative grade** | | |
| High | 85/88, 96.6% (90.4–99.3) | - |
| Medium | 50/53, 94.3% (84.3–98.8) | - |
| Low | 35/49, 71.4% (56.7–83.4) | - |
| Very low | 9/23, 39.1% (19.7–61.5) | - |
| Trace | 2/16, 12.5% (1.6–38.3) | - |

Abbreviations: CI: confidence interval.

**Table S11:** Diagnostic accuracy compared to MRS of combined tongue swab and sputum MiniDock MTB test results*

| | **Sensitivity** n/N, % (95% CI) | **Specificity** n/N, % (95% CI) |
|---|---|---|
| **MiniDock MTB** | 198/226, 87.6% (82.6–91.6) | 1037/1067, 97.2% (96–98.1) |

* The combined result was considered positive if either test was positive, and negative only if both tests were negative.
Abbreviations: MRS: microbiological reference standard, CI: confidence interval.

**Table S12:** Usability study participant characteristics

| Characteristics | n | % |
|---|---|---|
| **Country** | | |
| India | 10 | 55.56 |
| South Africa | 8 | 44.44 |
| **Occupation** | | |
| Nurse | 2 | 11.11 |
| Laboratory technician | 6 | 33.33 |
| Medical doctor/clinical officer | 10 | 55.56 |
| | **Median** | **IQR** |
| **Age, years** | 29 | 28 – 33 |
| **Overall experience, years** | 6 | 3 – 10 |
| **Experience working on TB, years** | 3 | 1 – 5 |

Abbreviations: IQR: inter-quartile range, TB: tuberculosis

**Table S13:** Summary of task completion and observed error rates during usability evaluation of MiniDock MTB

| Task | Description | Completion n/N (%) | Error n/N (%) |
|---|---|---|---|
| 1 | Prepare the testing device | 18/18 (100%) | 2/18 (11.1%) |
| 2 | Verify kit components and expiration date | 14/18 (77.8%) | 6/18 (33.3%) |
| 3 | Tongue swab collection: Ask and ensure that the simulated patient has not coughed, brushed teeth, rinsed mouth, used tobacco or consumed food/drink in the past 30 minutes | 15/18 (83.3%) | 3/18 (16.7%) |
| 4 | Collect tongue swab sample | 17/18 (94.4%) | 5/18 (27.8%) |
| 5 | Sputum collection simulation: Instruct simulated patient to rinse mouth | 8/18 (44.4%) | 13/18 (72.2%) |
| 6 | Immerse the swab in simulated sputum sample, swirl 10 times for 10–15 seconds and remove from container | 15/18 (83.3%) | 6/18 (33.3%) |
| 7 | Remove seal of the Nucleic acid releasing agent 02 | 18/18 (100%) | 1/18 (5.6%) |
| 8 | Insert swab into the vial twist and pinch swab tip against the bottom of the vial 10 times | 18/18 (100%) | 4/18 (22.2%) |
| 9 | Remove, dispose swab, screw tubes cap | 18/18 (100%) | 3/18 (16.7%) |
| 10 | Insert the tube in the Thermolyse or constant temperature mixer | 17/18 (94.4%) | 7/18 (38.9%) |
| 11 | Press RUN/STOP to begin the lysis step and wait 5 minutes | 16/18 (88.9%) | 5/18 (27.8%) |
| 12 | Connect MiniDock to a power supply | 17/18 (94.4%) | 3/18 (16.7%) |
| 13 | Turn on MiniDock device | 17/18 (94.4%) | 1/18 (5.6%) |
| 14 | Open the reaction card, place in holder | 17/18 (94.4%) | 5/18 (27.8%) |
| 15 | Apply releasing agent to reaction card | 18/18 (100%) | 6/18 (33.3%) |
| 16 | Screw cap on reaction card | 18/18 (100%) | 2/18 (11.1%) |
| 17 | Press top air bag to deform sample into tube | 16/18 (88.9%) | 8/18 (44.4%) |
| 18 | Observe the bubble volume and make sure it occupies no more than 1/3 of chamber of reaction card | 13/18 (72.2%) | 5/18 (27.8%) |
| 19 | Hold the chip and shake it up and down 10 times in about 5 seconds | 18/18 (100%) | 6 /18 (33.3%) |
| 20 | Load Reaction Card into the device and close cover | 18 /18 (100%) | 4/18 (22.2%) |
| 21 | Press power button to start detection and wait without opening the door of the device | 18/18 (100%) | 0/18 (0%) |
| 22 | Dispose of used components | 18/18 (100%) | 0/18 (0%) |

**Table S14:** Summary of use errors during MiniDock MTB testing and the potential risks associated with the errors.

| Severity | Issue or error | Description | Potential risk to safety and/or validity |
|---|---|---|---|
| **Critical** | Omission of lysis step | One participant skipped the lysis step entirely and completed the test without recognizing the omission. Another attempted to open the device mid-process to correct this oversight. | High risk of invalid or inaccurate results due to incomplete lysis; critical for test validity. |
| **Critical** | Overfilling reaction card | One participant held the card in reverse, obscuring the indicator lines. Others failed to observe the lines and overfilled the card. | May alter reaction conditions and compromise result accuracy; error is not easily correctable once made. |
| **Major** | Expiration date not verified | Several participants did not check kit expiration dates prior to use. | Potential use of expired or degraded materials, increasing risk of invalid or inaccurate results. |
| **Major** | Omission of sputum pre-collection mouth rinse | Some participants did not instruct the (simulated) patient to rinse their mouth before sputum collection. | Risk of sample contamination, affecting test validity. |
| **Major** | Incorrect placement of reaction card | One participant placed the card into the MiniDock instead of the cardholder, triggering premature QR code scanning. | May disrupt workflow and cause misalignment or system errors. |
| **Minor** | Hesitation due to IFU layout | Participants displayed non-verbal sings of confusion and hesitation when navigating the IFU. | May slow workflow but does not directly affect result validity or safety. |

**Table S15:** Summary of qualitative findings on usability and preliminary acceptability of MiniDock MTB.

| Themes | Summary of Findings | Illustrative Quote |
|---|---|---|
| Usability | | |
| Pluslife Thermolyse operation | Participants showed confusion due to a lack of diagrams, unlabeled buttons, and insufficient IFU instructions. | *"[The IFU] needed more pictures. [There were] needed pictures of materials used for testing. Thermolyse image needed in IFU."* - Participant from India |
| | Types of critical errors observed included skipping the Thermolyse step, incorrect operation (e.g., opening the device mid-process), and failure to pre-warm the device. | *"I wasn't sure what the Pluslife Thermolyse was, how to set it up and insert the required values."* - Participant from South Africa<br>*"All the steps are clear except Thermolyse step."* - Participant from India |
| | Participants requested clearer visual guidance and improved graphical representation in the IFU. | *"Maybe guide on how to use the Thermolyse, just as this was done for the MiniDock."* - Participant from South Africa<br>*"[Having] a bit more details regarding the Thermolyse could be helpful."* - Participant from India |
| Reaction card handling | Participants struggled with proper handling and card orientation. Some held the card incorrectly, risking contamination or overfilling. | *"[I found difficult] the step, press the protruding arc shaped air bag on sample tube cap of reaction card to deform it and recess it into the tube."* - Participant from India |
| | One participant held the card in reverse, obscuring indicator lines and causing overfilling. | *"The card holder isn't mentioned to use for pressing down of the cap… bubbles in the slide are difficult to interpret."* - Participant from South Africa |
| | Errors were attributed to poor contrast and lack of visual guidance in the IFU. In some cases, materials had to be replaced. | *"It seemed very important to aliquot… but the lines on the card were hard to see."* - Participant from South Africa |
| MiniDock device | One participant misidentified the MiniDock as the cardholder, triggering premature QR code scanning. | […] *"the card holder isn't mentioned to use for pressing down of the cap. I ended up using the machine as the card holder."* - Participant from South Africa |
| Acceptability | | |
| Usability and workflow | The test was described as easy to use, with an intuitive workflow and minimal training requirements. Several participants found it easier than traditional TB diagnostics like smear microscopy. | *"Any person with the manual can easily perform the tongue swab test whereas with the sputum smear microscopy needs hands on training."* - Participant from South Africa |

| | | |
|---|---|---|
| Sample collection and processing | Participants appreciated the tongue swab for being non-invasive and suitable for patients unable to expectorate sputum. | *"[The test is] Very quick and easy to do. Tongue swab would be easier to collect and less stigma…"* - Participant from India |
| | Tongue swab collection was viewed as simpler and less demanding than sputum collection, which involve additional processing steps like liquefaction. | *"The sputum swab test is easier and more convenient to use compared to the sputum Xpert Ultra, specifically related to the liquification of the sputum in Xpert Ultra."* - Participant from South Africa |
| Portability and point-of-care use | Participants saw portability and quick results as advantageous for use in primary care and low-resource settings. | *"This test can be implemented in primary care centers since the machine is portable."* - Participant from India |
| | While a power bank was used during the evaluation, reliance on electricity was noted as a limitation for some settings. Battery operation was suggested. | *"The tongue swab test apparatus would require steady electricity supply which might not be available…"* - Participant from South Africa |
| Instructions for Use (IFU) | Participants suggested clearer and more comprehensive visuals in the IFU to assist less experienced users. | *"[It] would be helpful to have scientific names of instruments color coded and matched with a color code in the diagram for the Pluslife Thermolyse apparatus."* - Participant from South Africa<br>*"[I] would like to include more diagrammatic representation so that everyone can use [it]."* - Participant from India |
| Concerns and Limitations | Some participants raised questions about cost, diagnostic scope, and biosafety during sample collection | *"I am not sure of the cost but I think the tongue swab test might be more expensive."* - Participant from South Africa<br>*"Sensitivity results will not be available for Rifampicin."* - Participant from India<br>*"The gag reflex can cause patient to cough in your face."* - Participant from South Africa |

# References


1. Lumb R, Van Deun A, Bastian I, Fitz-Gerald M, Pathology SA, Stop TB Partnership (World Health Organization). Laboratory diagnosis of tuberculosis by sputum microscopy: the handbook. 2013.

2. Salman H Siddiqi, Sabine Rüsch-Gerdes. MGIT Procedure Manual for BACTEC™ MGIT 960™ TB System [Internet]. 2006. Available from: https://www.finddx.org/wp-content/uploads/2023/02/20061101_rep_mgit_manual_FV_EN.pdf

3. In vitro diagnostic medical devices used for the qualitative detection of Mycobacterium tuberculosis complex DNA and mutations associated with drug-resistant tuberculosis [Internet]. Geneva: World Health Organization; 2022. Available from: https://apps.who.int/iris/bitstream/handle/10665/366068/9789240055865-eng.pdf

4. Carpenter JR, Smuk M. Missing data: A statistical framework for practice. Biometrical Journal 2021;63(5):915–47.
